# Electrically Tunable Lenses: A Review

**DOI:** 10.3389/frobt.2021.678046

**Published:** 2021-06-09

**Authors:** Leihao Chen, Michele Ghilardi, James J. C. Busfield, Federico Carpi

**Affiliations:** ^1^ School of Engineering and Materials Science, Queen Mary University of London, London, United Kingdom; ^2^ Department of Industrial Engineering, University of Florence, Florence, Italy

**Keywords:** electrical, tunable, lens, liquid, elastomer, silicone, soft, deformable

## Abstract

Optical lenses with electrically controllable focal length are of growing interest, in order to reduce the complexity, size, weight, response time and power consumption of conventional focusing/zooming systems, based on glass lenses displaced by motors. They might become especially relevant for diverse robotic and machine vision-based devices, including cameras not only for portable consumer electronics (e.g. smart phones) and advanced optical instrumentation (e.g. microscopes, endoscopes, etc.), but also for emerging applications like small/micro-payload drones and wearable virtual/augmented-reality systems. This paper reviews the most widely studied strategies to obtain such varifocal “smart lenses”, which can electrically be tuned, either directly or via electro-mechanical or electro-thermal coupling. Only technologies that ensure controllable focusing of multi-chromatic light, with spatial continuity (i.e. continuous tunability) in wavefronts and focal lengths, as required for visible-range imaging, are considered. Both encapsulated fluid-based lenses and fully elastomeric lenses are reviewed, ranging from proof-of-concept prototypes to commercially available products. They are classified according to the focus-changing principles of operation, and they are described and compared in terms of advantages and drawbacks. This systematic overview should help to stimulate further developments in the field.

## Introduction

Research on electrically tunable optical lenses has been growing in the past couple of decades. The main motivation is a reduction of the complexity, size, weight, response time and power consumption of conventional focusing/zooming systems. Indeed, the latter are based on glass lenses that are translated using electromagnetic or electrostatic motors. Conversely, an electrically tunable lens is here referred to as a refractive medium having a focal length that can dynamically be tuned, without any mechanism that shifts the lens plane.

Such tunable lenses might become especially relevant for a diversity of robotic and machine vision-based devices. Whilst cameras for portable consumer electronics (e.g. smart phones) and advanced optical instrumentation (e.g. microscopes, endoscopes, etc.) are evident examples, there are also other and less obvious potential applications. One of them is, for instance, small/micro-payload drones (flying, legged, floating, etc.), whose push toward miniaturization and autonomy extension is going to challenge even their cameras, in terms of size, weight, speed and energy efficiency, not only for robotic inspection and monitoring tasks, but also for embodied telepresence as first-person-view flights and explorations (Jablonowski, 2020). Another example is wearable tunable optics for virtual/augmented-reality systems, which also need compact, light-weight, fast and low power consuming, as well as silent, lenses for zooming and focusing (Stevens et al., 2017; Wang and Lin, 2019).

So far, a broad diversity of tunable lenses has been described (Ren and Wu, 2012; Zappe and Duppé, 2016). Previous systematic reviews have covered so-called liquid or optofluidic lenses (Levy and Shamai, 2008; Nguyen, 2010; Chiu et al., 2012; Mishra et al., 2016; Huang and Zhao, 2019), consisting of liquids or, more generally, fluids (including also gels), encapsulated within rigid or deformable enclosures.

Here, we extend the scope, including also fully elastomeric tunable lenses and covering recent advances on both kinds. Moreover, a systematic classification of all the available technologies, according to their working principles, is proposed, so as to simplify comparisons. The underling general approaches are described and compared in terms of pros and cons.

It is worth noting that this Review aims at covering only strategies that ensure controllable focusing of multi-chromatic light, with spatial continuity (i.e. continuous tunability) in wavefronts and focal lengths, as required for visible-range imaging applications. For instance, Fresnel lenses and spatial light modulators will not be discussed. Indeed, they typically degrade the image quality, due to step-like wavefront modulation; so, even if they might be usable, as an example, for basic sensing (e.g. object recognition) in computer vision, they currently are not attractive for imaging. Similarly, the discussion will not include metamaterial lenses. Indeed, whilst they are now able to capture usable images (Zou et al., 2020), they typically show significant chromatic aberrations; although achromatic versions have been demonstrated (Aieta et al., 2015; Chen et al., 2018), they cannot be implemented (at least not yet) in combination with focal length tuning. In fact, whilst metamaterial lenses with electrical tunability have been reported (She et al., 2018), they are still challenged by chromatic aberrations.

In this Review, the presentation of the topics intentionally avoids excessive technicalities, in order to offer a simple guide to navigate the landscape of these tunable optical devices, even for readers that have no expertise in optics or in the associated implementing technologies.

## Focal Length Tuning Strategies

An electrically tunable lens, or “smart lens”, is here defined as a light focusing medium (in the form of either a fluid, a gel or a solid) that is able to use an input electrical energy to dynamically modulate its focal length. Therefore, in a tunable lens it is possible to modify, by electrical means, some internal properties that change the shaping of the light wavefront.

The main strategies described so far to achieve that goal for multi-chromatic light in the visible range can be divided into two main groups, which differ according to the physical property modified by the input electrical energy: the lens’ refractive index or the lens’ shape (surface curvature). The second approach is exploited by the vast majority of tunable lenses developed so far.

**FIGURE 1:**
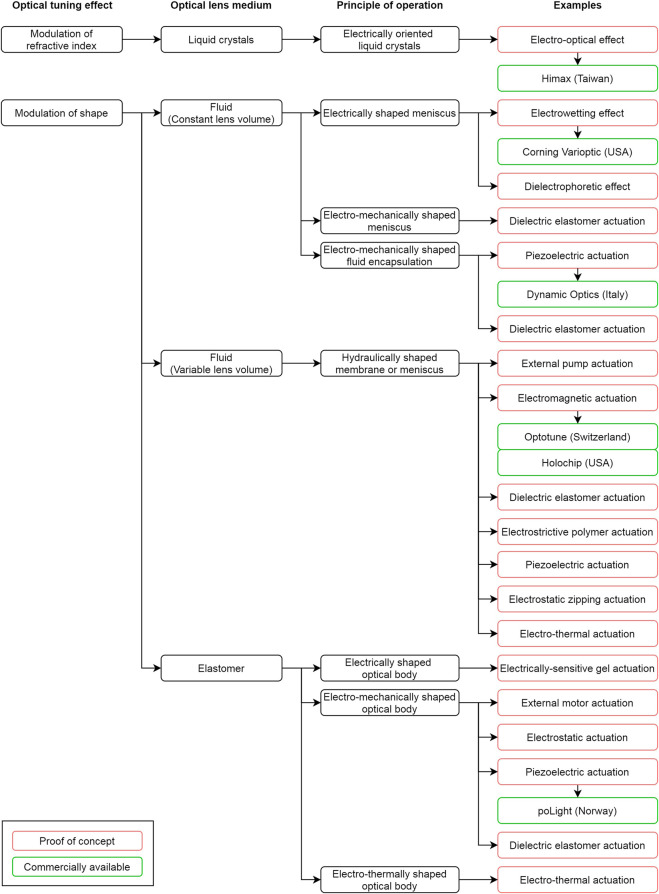
Proposed classification of electrically tunable lenses operating in the visible range according to a modulation of either their refractive index or their shape (surface curvature). This classification intentionally covers only principles of operation that enable a controllable focusing of multi-chromatic light, with spatially continuous (i.e. continuously tunable) wavefronts and focal lengths.

Among those strategies, some tune the focal length upon direct electrical driving, while others via electro-mechanical or electro-thermal coupling. All the approaches are classified in Figure 1, according to the controlled physical property, the constitutive matter of the main optical medium (liquid crystals, fluids or elastomers) and the principle of operation.

Each type of tunable lens is described below, with reference to significant examples, consisting of research prototypes and/or commercial products. For each technology, the most relevant pros and cons are discussed.

## Liquid Crystal Tunable Lenses

Liquid crystal (LC) tunable lenses work according to an electro-optical effect, i.e. a change in optical properties of a material, in response to an applied electric field. In particular, common LCs are rod-like molecules (so-called nematics) (Yang and Wu, 2014), not only having predictable molecular orientations like crystals, but also featuring fluidity like liquids. They typically have a gel-like state, although they can also be solidified (film-like, when polymerized). The orientations of LCs can be controlled by an electric field. This can be used to spatially and dynamically change their effective refractive index for a polarized light under a certain angle of incidence. Since the 1970s, this effect has been exploited to obtain electrically tunable lenses (Sato, 1979).

**FIGURE 2:**
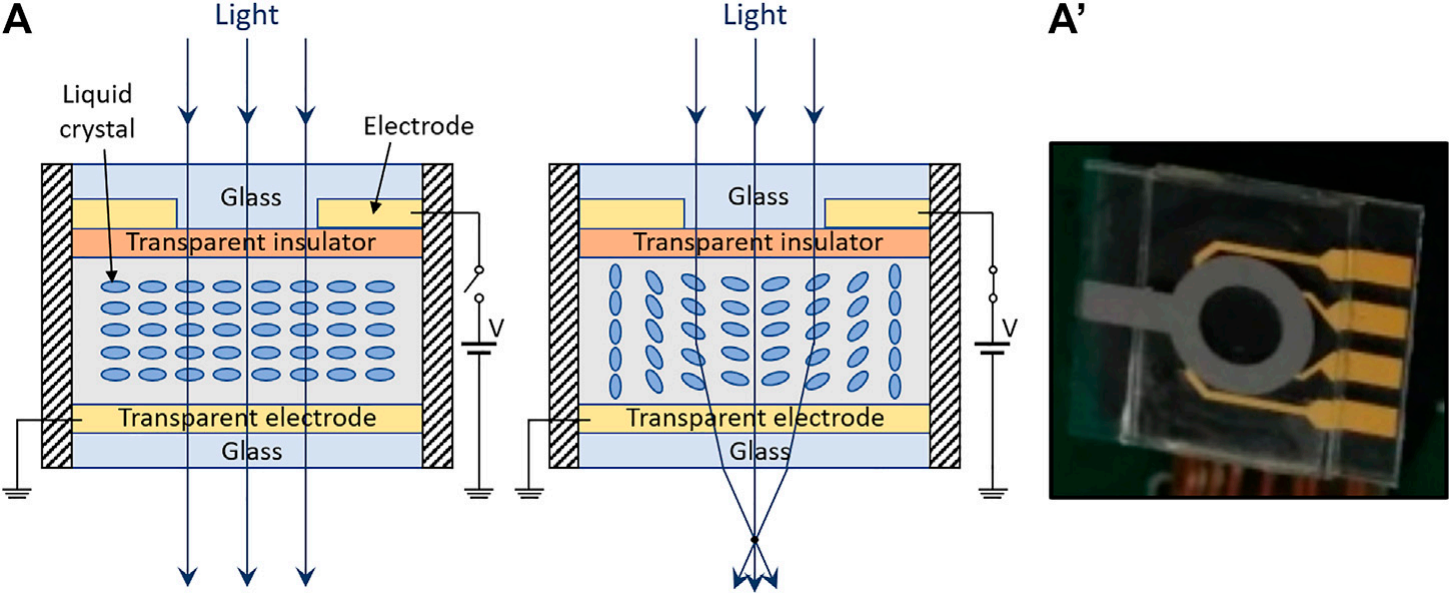
Liquid crystal lens. **(A)** Schematic of a possible structure (taken as an example among various alternatives) and related principle of operation: an applied voltage creates a non-uniform electric field, which spatially varies the orientation of liquid crystals, causing a non-uniform refractive index and, so, a light focusing effect; **(A′)** Prototype sample, reproduced with permission from (Allen, 2014).

Whilst a variety of structures has been used to obtain various kinds of LC lenses, essentially each of them consists of a LC medium, where an incident plane wave of light is focused by an electrically controllable distribution of LC directors (Naumov et al., 1998). In particular, in so-called GRIN (GRadient INdex) LC lenses, by using an appropriate electrode arrangement to apply a non-uniform electric field, a polarization-dependent spatial profile of the refractive index is generated. This is used to focus light, such that the focal length can be modulated from optical infinity to close distance. Figure 2A shows an example of a structure, among a variety of possible alternatives (Lin et al., 2017).

LC lenses are commercially available for instance from the company Himax, which offers lenses with an aperture of 6 mm and a response time lower than 2 s (Himax, 2021). Another company, LensVector, also commercializes LC lenses, although at present they serve for light shaping (modulation of illumination) rather than for imaging (LensVector, 2021).

In general, LC lenses advantageously require relatively low driving voltages (order of 10–100 V) and have low power consumption (Lin et al., 2017). Moreover, their optical profile can also be made aspherical (Lin et al., 2017), which is useful to correct various aberrations.

As a drawback, their response time is temperature dependent and it also increases with the LC layer’s thickness, typically reaching the order of 10–100 ms (Lin et al., 2017). However, lowering the thickness is not entirely beneficial, as thicker layers allow for an extended tuning range, as the minimum focal length is inversely proportional to the thickness (Lin et al., 2017). So, LC lenses typically require a trade-off between the response time and the tuning range.

As a consequence, at present dynamic imaging does not seem to be a suitable field of application for LC lenses. Nevertheless, they look promising as a low-speed but also low-voltage technology for wearable tunable optics, such as spectacles for correction of presbyopia (Jamali et al., 2020).

Another typical drawback of LC lenses is their limited aperture. Indeed, the minimum focal length (and so also the tuning range) is proportional to the square of the aperture (Lin et al., 2017). So, LC lenses require also a trade-off between the aperture and the tuning range.

A variety of strategies is being explored to overcome such limitations. For instance, Lin et al. (2010) demonstrated a lens with a response time of ∼433 ms and a large tuning range, from 300 to 10 cm, as a result of a combination of three factors: a reduction of the LC layer’s thickness (25 μm), the application of relative high voltages (90 V_rms_ i.e. ∼127 V) and a switching between positive and negative lens modes (Lin and Lin, 2010).

Frequently, as an additional limitation, LC lenses need a polarizer, as they work with polarized light. Nevertheless, in recent years, significant efforts have been spent to avoid the dependence on polarization, so as to have polarizer-free lenses. Among a diversity of possible strategies (Lin et al., 2017), one of the most used is represented by stacking multiple LC layers having different alignments of the optical axes (Chen et al., 2015; Kumar et al., 2020). However, at present, polarizer-free, continuously tunable LC lenses have apertures that do not exceed the order of magnitude of 10 mm (Lin et al., 2017; Jamali et al., 2020).

It is worth noting that, in addition to GRIN-type LC lenses discussed above, another kind of LC lenses is emerging. They are referred to as polarization-dependent (or polarization-directed) flat lenses, also knowns as waveplate lenses or Pancharatnam-Berry phase lenses. They consist of a thin flat window coated with a LC polymer film, which forms a grating that is sensitive to incident light having a circular polarization: depending if the latter is left- or right-handed, the lens can show a positive or negative focal length. The two focal lengths can electrically be switched, by combining the lens with a controllable phase retardation plate, which changes the polarization of light (Gao et al., 2015; Tabiryan et al., 2015). This type of lens is produced for instance by the company Beam Co. (Beam Co, 2021). In order to use these “bifocal lenses” to modulate a focal depth across a certain range of distances (as required for imaging purposes), it is necessary to stack many of them and individually control each of them. So, with a stack of N lenses, it is possible to sweep through 2^N^ focal planes. For instance, this has been shown with a prototype varifocal lens for an Oculus virtual-reality headset (Blog, 2019). The main advantage of these lenses is that they are thin and flat. However, their focal length cannot be modulated with continuity and, as they are gratings, they typically have significant chromatic aberration.

## Fluid-Based Tunable Lenses With Constant Volume

### Electrically Shaped Meniscus Lenses

A tunable focusing of light can be achieved by electrically controlling the curvature of a meniscus between two immiscible liquids. This effect is exploited in so-called “electrowetting lenses” and “dielectrophoretic lenses”, which are separately presented below.

#### Electrowetting Effect

Electrowetting lenses represent the most studied technology using an electrical controllability of a meniscus. Electrowetting in general refers to the change of contact angle of a conductive liquid droplet interfaced to a solid substrate (within a liquid or gaseous environment), in response to an applied electric field (Mugele and Baret, 2005).

**FIGURE 3:**
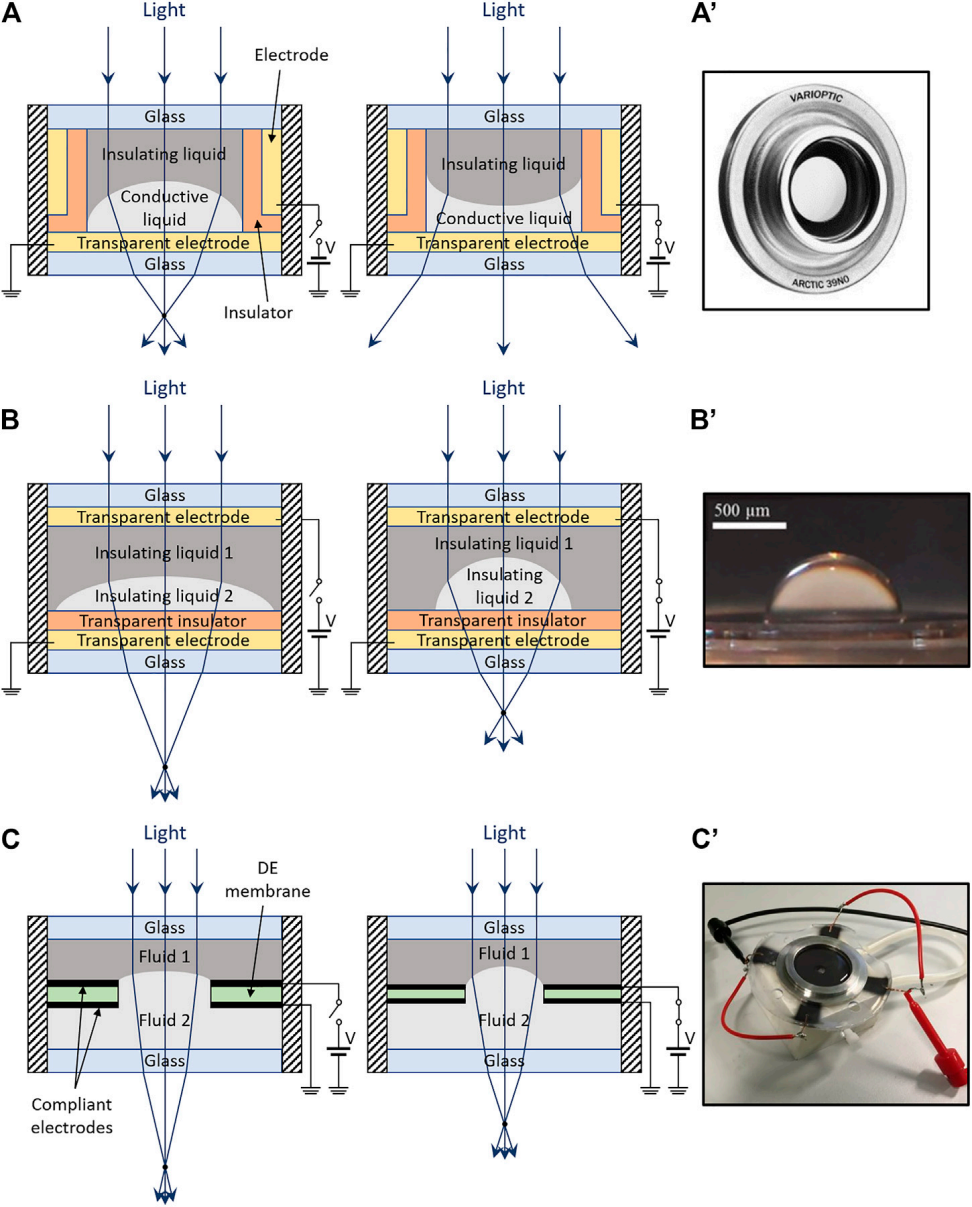
Electrically or electro-mechanically shaped meniscus lenses. Structures and principles of operation **(left)** and photos of examples **(right)**, for different actuation strategies: **(A)** schematic of an electrowetting lens (example among various alternatives), where an applied electric field changes the contact angle of a conductive liquid droplet; **(A′)** commercial product by Corning Varioptic, adapted from (Varioptic, 2021); **(B)** schematic of a dielectrophoretic lens (example among various alternatives), where an applied electric field changes the contact angle of an insulating liquid droplet; **(B′)** prototype sample, reproduced with permission from (Almoallem and Jiang, 2017); **(C)** schematic of an electro-mechanically controlled meniscus lens, where the curvature of a meniscus is changed by an electrically induced deformation of an annular DE actuator; **(C′)** prototype sample, reproduced with permission from (Rasti et al., 2015b).

Liquid tunable lenses based on the electrowetting effect are a relatively young technology (Berge and Peseux, 2000). They are made of a conductive liquid droplet (acting as a plano-convex lens), in contact with both a solid substrate and an immiscible insulating liquid, as schematically shown in Figure 3A. By applying a voltage between the conductive liquid droplet and an electrode coated with an insulating layer on the substrate, the contact angle between the droplet and the substrate varies; the resulting change of curvature of the droplet’s surface modifies the focal length (Berge and Peseux, 2000; Kuiper and Hendriks, 2004; Hendriks et al., 2005; Mugele and Baret, 2005). A commercial version of these lenses is shown in Figure 3A’.

As an example of performance, a 300 μm-aperture lens was reported to vary its focal length from 2.3 mm to optical infinity at 45 V (Krogmann et al., 2006). DC driving advantageously enables low power consumption (Krogmann et al., 2006; Watson et al., 2015), as it is due to only leakage currents. However, prolonged exposure to DC voltages can lead to accumulation of charges, challenging the reversibility of the effect and causing hysteresis, such that AC driving is frequently preferred (Watson et al., 2015).

In addition to a tunable focal length, Kopp and Zappe (2016) demonstrated the first electrowetting lens with tunable astigmatism, along four directions; it had an aperture of 3 mm and was driven at ∼50 V_rms_.

A reduced operating voltage was demonstrated by Watson et al. (2015), using insulators made of parylene thin films, which allowed for the insulation thickness to be reduced from the (typical) order of 1 μm to a few 100 nm; in particular, the focal length of a 2.7 mm-aperture lens changed from about −23 mm to optical infinity at 15 V.

Most electrowetting lenses enable both convex and concave lens profiles (hereinafter indicated with respectively positive and negative focal lengths) and offer large tuning ranges. This is typically made possible by using insulations that can sustain increasing voltages, such that the lens profile at a certain critical voltage switches from convex to concave. For instance, Li and Jiang (2012) described a 4 mm-aperture flexible lens capable of varying its focal length from 15 mm to optical infinity at ∼115 V, and then down to −28 mm at 145 V, with a response time of ∼50 ms. In Watson et al. (2015), the focal length changed from 23 mm to optical infinity at 15 V, and then to −100 mm at 30 V.

A different approach to enable both convex and concave lens profiles was described by Li et al. (2019a): an electrowetting lens chamber was combined with an electrowetting actuation chamber, so as to modulate the amount of liquid contained in the former.

Electrowetting lenses are commercially available, with different apertures (order of 1–10 mm), as Corning^®^ Varioptic^®^ lenses (Varioptic, 2021). As an example, in some models the focal length can be changed from ∼56 mm up to optical infinity at ∼38 V, and then to −33 mm at 55 V (Varioptic, 2021). Their response time (time to reach 90% of the response) can be as low as ∼10 ms (Liu et al., 2008). In general, electrowetting lenses have a compact structure and require voltages of the order of 10–100 V.

Due to the liquid nature of the interface, a limitation of electrowetting lenses is a possible sensitivity to mechanical shocks/vibrations (Yu et al., 2012) and to gravitational sagging (which, if it is to be avoided, requires the liquids to be of equal density).

These effects also limit the maximum aperture. Additionally, the aperture is limited even by the need for reducing the volume of liquids to be displaced, so as to reduce both the response time (to an applied voltage) and the recovery time (needed by the liquid interface to regain its original shape after the voltage is removed). Typically, the aperture is restricted to a few millimetres (Watson et al., 2015; Varioptic, 2021).

The response time (which, for any given aperture, depends on the density and viscosity of the liquids, as well as on the surface tension between them) is in general of the order of 10–100 ms.

A shaping of the driving voltage signal has been shown to be critical in order to identify, as expected, an optimal trade-off between a desirable high response speed and a disadvantageous low damping of the fluid interface’s oscillations that are induced by the electrical stimulus (Supekar et al., 2017).

Another limitation is sensitivity to thermal fluctuations, whose effects on the liquids can alter the lens profile; indeed, open-loop driving requires a temperature sensor to enable compensations (Varioptic, 2021).

#### Dielectrophoretic Effect

Another technology that relies on electrical modulations of a meniscus is known as dielectrophoretic lenses, also referred to as dielectric liquid lenses (Cheng and Andrew Yeh, 2007; Ren et al., 2008). They use dielectrophoresis, which can be defined as a displacement of neutral matter caused by polarization, induced by a non-uniform electric field (Pohl, 1978).

Dielectrophoretic lenses have a structure similar to that of electrowetting lenses, as they both contain two immiscible liquids; however, in dielectrophoretic lenses, both liquids are insulating, with different dielectric constants, as schematically shown in Figure 3B. By applying an non-uniform electric field, the droplet experiences a dielectric force, which deforms it, according to the field gradient and the dielectric constant difference with the surrounding liquid (Cheng and Andrew Yeh, 2007; Ren et al., 2008). The resulting change of contact angle with the substrate causes a focal length variation. An example of this type of lens is shown in Figure 3B’.

The required field gradient within the droplet can be created in different ways. One method is to use parallel-plate continuous electrodes (as in Figure 3B) and exploit the different confinement of the field within adjacent portions of the droplet having a different height, given the difference of dielectric constant with the other liquid (Ren et al., 2008). Another method is to use patterned electrodes, for instance via an array of concentric rings (Cheng and Andrew Yeh, 2007).

As for electrowetting lenses, even dielectrophoretic lenses can use DC driving (which is convenient in terms of low power consumption), although AC driving is frequently implemented to avoid accumulation of charges.

Moreover, as for electrowetting lenses, larger apertures typically correspond to higher response times, due to larger volumes of fluid to be displaced. For instance, Cheng and Andrew Yeh (2007) described a 3 mm-aperture lens with a focal length tunability from 34 to 12 mm at 200 V, with a rise time of 650 ms and a fall time of 300 ms. A more challenging outcome was obtained by Jin et al. (2017), who reported for a 3 mm-aperture lens a focal length change from ∼19.3 to ∼1.5 mm at 40 V_rms_, with a rise time of 700 ms and a fall time of 6,000 ms. A microlens with a 230 µm-aperture was reported by Ren et al. (2008), showing a rise time of ∼200 ms and a fall time of ∼200 ms, for a change in focal length from 620 to 500 μm at 90 V_rms_. In general, electrowetting lenses have response times (rise times) of the order of 100–1,000 ms.

The driving voltages can be reduced with electrodes having spatial arrangements that magnify the electric field gradient: for instance, a variation of focal length from 67.1 to 14.4 mm at 25 V_rms_ was demonstrated for a 1 mm-aperture lens by Almoallem and Jiang (2017).

In general, dielectrophoretic lenses share with the electrowetting ones operating voltages of the order of 10–100 V and low power consumption.

They also share the same limitations. So, their aperture is typically limited to a few millimetres, owing to a sensitivity to mechanical shocks/vibrations and gravitational sagging, as well as a need to limit the amount of fluid, so as to reduce the rise and recovery times. Moreover, even dielectrophoretic lenses are sensitive to thermal fluctuations (Zhang et al., 2014). Nevertheless, dielectrophoretic lenses advantageously avoid the risk for electrolysis, Joule heating and formation of microbubbles, which can arise while charging conductive liquids (Xu et al., 2013).

### Electro-Mechanically Shaped Meniscus Lenses

Modulations of the shape of a meniscus between immiscible liquids have also been achieved with electro-mechanical actuation. In particular, tunable lenses based on this concept have been demonstrated using dielectric elastomer (DE) actuation.

DE actuators essentially are electrically deformable capacitors, typically consisting of a DE membrane coated with two compliant electrodes; by applying a voltage between the electrodes (introducing an electric field across the membrane), an expansion in surface and a compression in thickness of the structure is obtained, due to a Maxwell stress (Pelrine et al., 1998; Pelrine et al., 2000).

A possible way to use DE actuation to operate a meniscus lens is shown in Figure 3C. It was described by Rasti et al. (2015a); Rasti et al. (2015b) and it comprises two immiscible liquids interfaced to an intermediate annular DE actuator; the two liquids form a meniscus at the actuator’s central hole. Electrically induced expansions of the DE actuator membrane are used to reduce the hole’s diameter, thereby shrinking the meniscus and increasing its curvature. Figure 3C’ shows a 4 mm-aperture silicone-made sample, capable of a focal length change from ∼190 to ∼40 mm at 600 V (Rasti et al., 2015b).

While this solution is expected to offer high tuning speeds (although no data are available so far), it is intrinsically limited to relatively small apertures, as for the other types of meniscus-based lenses. Moreover, at present, the use of DE actuation requires high driving voltages (although at low electrical powers), which cannot be reduced below a few hundred Volts, even by stacking multiple membranes with multiple electrodes pairs in electrical parallel (Rasti et al., 2015a; Rasti et al., 2015b).

### Electro-Mechanically Shaped Encapsulated-Fluid Lenses

A different family of fluid-based tunable lenses having a constant volume consists of compliant structures, where a fluid is encapsulated by deformable membranes, made of elastomers or thin glass. Tunability is achieved via an actuation technology that deforms the whole enclosure. Piezoelectric and DE actuators are the most used electro-mechanical transducers adopted for that purpose, as presented below.

#### Piezoelectric Actuation

**FIGURE 4:**
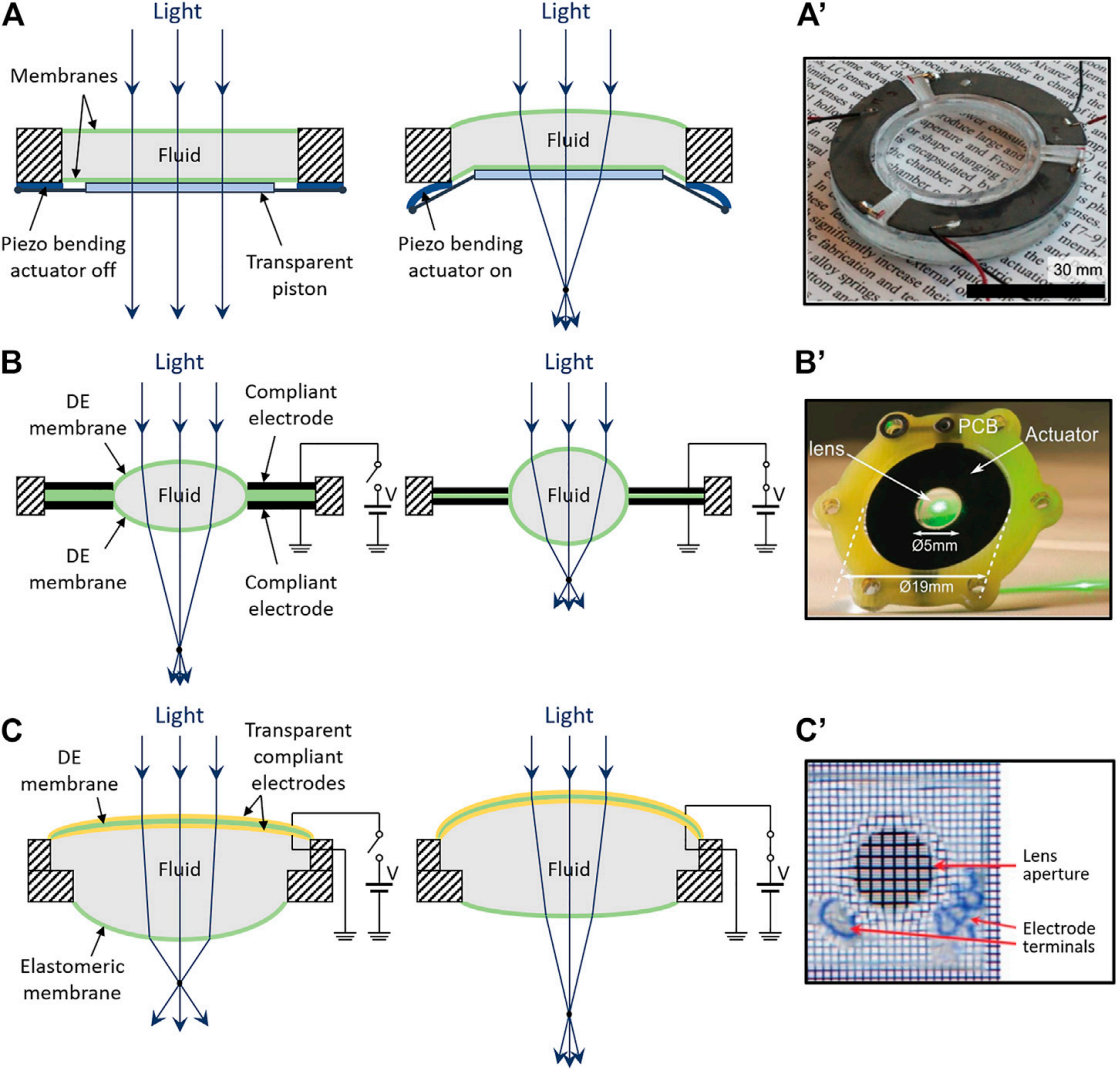
Electro-mechanically shaped encapsulated-fluid lenses. Structures and principles of operation **(left)** and photos of examples **(right)**, for different actuation strategies: **(A)** schematic of a lens based on piezoelectric bending actuators, which translate a piston that displace the fluid; **(A′)** prototype sample, reproduced with permission from (Hasan et al., 2017); **(B)** schematic of a lens based on an annular DE actuator, which radially compresses the central lens; **(B′)** prototype sample, reproduced with permission from (Maffli et al., 2015); **(C)** schematic of a lens based on a transparent DE actuator embedded on the surface, which increases its curvature; **(C′)** prototype sample, reproduced with permission from (Shian et al., 2013).

A piezoelectric-actuated tunable lens is sketched in Figure 4A. It was described by Hasan et al. (2017) and it consists of a transparent fluid encapsulated by two elastomeric membranes, one of which is connected to a rigid transparent piston. Piezoelectric bending actuators are connected to the piston, so that it can be moved to displace the fluid, and, therefore, change the free membrane’s curvature, varying the focal length. The use of bimorph benders enables bidirectional motions, so as to achieve plano-convex or plano-concave shapes of the lens.

Using polydimethylsiloxane (PDMS) membranes, glycerol as a fluid and three PZT-made bimorph benders, Hasan et al. (2017) assembled the prototype lens shown in Figure 4A’: with an aperture of 32 mm, it exhibited a tuning range between –493 and 280 mm for voltages between −250 and 250 V, with a power consumption of ∼20 mW and a response time of ∼15 ms.

A different kind of piezoelectric-actuated tunable lens was presented by Bonora et al. (2015). It comprised a fluid (mineral oil) encapsulated between a couple of thin (150 μm-tick) glass membranes; each membrane could be deformed by eight PZT-made bimorph bending actuators, driven at ±125 V. By activating different actuators groups, it was possible to deform different segments of the glass membranes. The resulting non-axial-symmetric modulation of the lens shape was used to vary not only defocus, but also astigmatism, coma and secondary astigmatism aberrations (Bonora et al., 2015).

A similar design was adopted by Peng et al. (2020), although in this case the bending piezoelectric actuators covered only one side of the lens (which had an aperture of 20 mm), and their number was increased to 32; independent driving of the actuators enabled modulations of low-order aberrations, such as defocus, astigmatism, trefoil and coma (Peng et al., 2020). Using actuators only on one side, Wapler (2020) demonstrated a 7.6 mm-aperture lens with a focal length range between about −143 and +167 mm, and a response time of ∼0.15 ms.

Piezoelectric-actuated fluid lenses are commercialized for instance by Dynamic Optics (Dynamic Optics, 2021). According to the company, those lenses can correct up to the fourth order of Zernike polynomial, with a response time lower than 5 ms and a focal length tuning from optical infinity to ∼286 mm (Dynamic Optics, 2021).

In general, a high tuning speed and control accuracy, in addition to low power consumption (due to a purely electrostatic driving), are the key advantages of piezoelectric-actuated lenses. However, the small electrically induced strains (order of 0.1%) of piezoelectric materials require strategies to magnify displacements, such as configurations as cantilever-type benders (as in Figure 4A), which occupy lateral space. Moreover, the limited displacements that in any case can be achieved with these mechanisms imply a limitation on the lens aperture (order of 1–10 mm), so as to ensure adequate variations of the lens curvature.

#### Dielectric Elastomer Actuation

A different strategy to electro-mechanically deform an encapsulated-fluid lens is to use DE actuation. A possible structure is presented in Figure 4B, which was described by Carpi et al. (2011). A fluid is encapsulated within a soft chamber, obtained by coupling two radially pre-stretched DE membranes. Electrical tunability is achieved by creating stretchable electrodes on the two sides of the coupled membranes around the lens, so as to obtain an annular DE actuator. When a voltage is applied between the electrodes, their expansion causes a radial compression of the lens, decreasing its radius of curvature and thus also its focal length.

Following a first demonstration with an acrylic elastomer (Carpi et al., 2011), PDMS was used by Maffli et al. (2015) as a material with lower viscoelastic losses to demonstrate faster response lenses (see a sample in Figure 4B’) with a response time as low as 175 μs; the lenses had an aperture of 5 mm and a focal length variation of −26% at ∼3 kV (Maffli et al., 2015).

A disadvantage of the design in Figure 4B is the lateral size of the actuation part. In order to avoid this limitation, Shian et al. (2013) proposed a distribution of DE actuation on the lens surface, rather than around it, as sketched in Figure 4C. This solution requires transparent compliant electrodes covering the inner and outer surfaces of one of the two membranes that form the lens; this way, one side of the lens behaves also as a transparent DE actuator: upon electrical charging, it increases its curvature, whilst the other side (which is not electroded) decreases it, owing to a fluid-mediated coupling. By implementing this strategy with acrylic elastomer membranes and carbon nanotube electrodes, Shian et al. (2013) demonstrated a lens (see Figure 4C’) capable of a focal length variation greater than 100% at 5 kV. The main advantage of this architecture is the compact size. Nevertheless, it challenges the lens transparency, owing to the presence of the electrode material along the optical path.

Similarly, Liang et al. (2014) used in-line transparent electrodes, consisting of a thin layer of gold on the outer side, and a NaCl solution, working also as the optical fluid, on the inner side.

Such investigations show the importance of stretchable transparent electrode materials for DE actuation-based lenses. Salty water is not a practically viable solution as an inner electrode, as the permeability to water of most DE membranes facilitates their electrical breakdown. Hydrogels as outer electrodes (Keplinger et al., 2013) are similarly not viable, as they tend to dry. A variety of choices that avoid volatile electrolytes is available, including electronic conductors, such as carbon nanotubes (Shian et al., 2013), graphene (Zang et al., 2013), polyethylenedioxythiophene - PEDOT (Son et al., 2012) and silver nano-wires (Shian and Clarke, 2016), as well as ionic conductors, such as non-volatile ionogels (Chen et al., 2014). Their achievable optical transmittance can vary, due to differences in composition and processing methods. So, the optical quality of tunable lenses based on transparent DE actuators is critically dependent on the quality of their electrodes.

In general, the most relevant pros of DE actuation-driven encapsulated-fluid lenses are fast responses and large apertures, as well as a small thickness and weight of the whole structure. However, they are all limited at present by the need for high driving voltages.

## Fluid-Based Tunable Lenses With Variable Volume

### Hydraulically Shaped Lenses

The broadest sub-group of fluid-based tunable lenses consists of deformable optical chambers containing a variable amount of fluid, which is displaced from/to a lateral reservoir, using a variety of actuation technologies. Therefore, such devices are shaped hydraulically. The final effect is a variation of the curvature of either a membrane sealing the chamber or a meniscus at the interface with a second immiscible fluid. Key examples are described below.

#### External Pump Actuation

**FIGURE 5:**
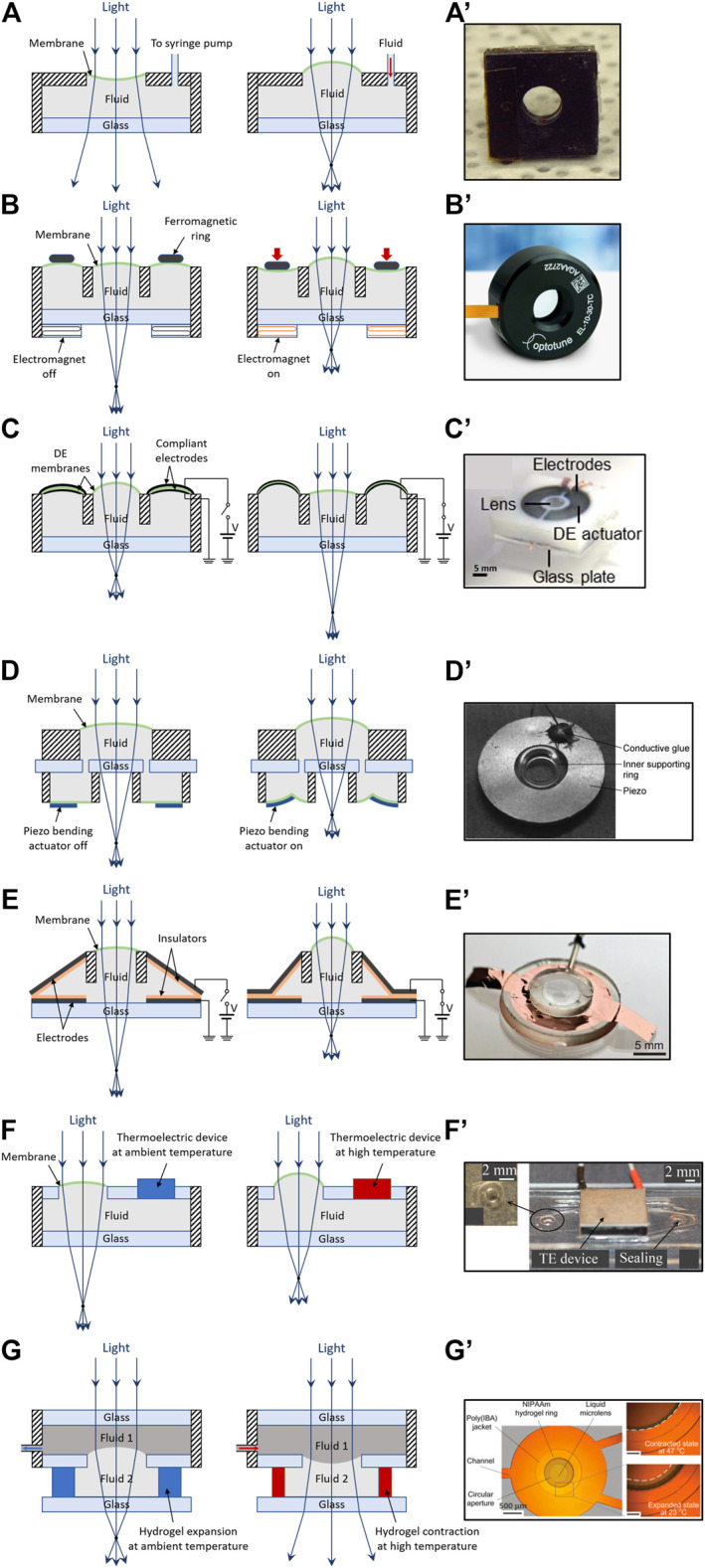
Hydraulically shaped lenses. Structures and principles of operation **(left)** and photos of examples **(right)**, for different actuation strategies: **(A)** schematic of a lens pressurized by an external pump; **(A′)** prototype sample, reproduced with permission from (Agarwal et al., 2004); **(B)** schematic of a lens pressurized by an electromagnetic actuator; **(B′)** commercial product by Optotune, adapted from (Optotune, 2021); **(C)** schematic of a lens depressurized by a toroidal DE actuator; **(C′)** prototype sample, reproduced with permission from (Wei et al., 2014); **(D)** schematic of a lens pressurized by a piezoelectric bending actuator; **(D′)** prototype sample, reproduced with permission from (Schneider et al., 2008); **(E)** schematic of a lens pressurized by an electrostatic zipping actuator; **(E′)** prototype sample, reproduced with permission from (Hartmann et al., 2020); **(F)** schematic of a lens pressurized by an electro-thermal expansion of the lens fluid; **(F′)** prototype sample, reproduced with permission from (Ashtiani and Jiang, 2013); **(G)** schematic of a lens meniscus deformed by an electro-thermal contraction of a polymer; **(G′)** prototype sample, reproduced with permission from (Dong et al., 2006).

The most straightforward strategy to obtain such tunable lenses is shown in Figure 5A: a fluid is pumped by an external unit into a chamber closed by a transparent elastomeric diaphragm (typically a PDMS membrane), acting as a lens surface with tunable curvature.

As an example, Zhang et al. (2003) used a syringe pump to tune the focal length of a 20 mm-aperture lens between 172 and 41 mm. Agarwal et al. (2004) used two diaphragms (one on each side) for a 4 mm-aperture lens (Figure 5A’) capable of biconvex and biconcave operation, showing a focal length variation from 75.9 to 3.1 mm and −75.9 to −3.3 mm, respectively. Lenses with apertures reduced to 0.4 mm were described by Werber and Zappe (2005).

Hydraulic driving has also been used to obtain tunable lenses made up of multiple independently controllable aligned chambers. This is useful, for instance, to compensate with a chamber an aberration created by a second chamber in the optical path. As an example, Waibel et al. (2011) showed a two-chambers lens with a focal length tuning between 5 and 40 mm and a chromatic aberration reduced by over 30%.

In general, whilst the use of external pumps enables large tuning ranges, it represents also the main limitation of this driving strategy, as it leads to systems that are bulky and inefficient.

#### Electromagnetic Actuation

Pumping systems to deform fluid-based lenses have been implemented even with more compact solutions, based on electro-magnetic driving. The magnetic field generated by an electro-magnet is used to attract a ferromagnetic body, which compresses a soft reservoir of the fluid.

An example is represented in Figure 5B: the attraction of a ring magnet compresses an elastomeric membrane, which covers an actuation chamber, so as to displace the fluid into a concentric lens chamber, where it varies the curvature of the lens membrane. Using this approach, Oh et al. (2016) demonstrated a ∼4 mm-aperture lens with rise and fall times of ∼900 ms each, which was capable of both concave and convex surface profiles, showing a focal length variation from −10 mm to optical infinity at ∼25 V, and then to 10 mm at 50 V.

A similar solution is used in commercial lenses (see Figure 5B’) produced by the company Optotune (Optotune, 2021). For instance, in their current-controlled EL-3-10 (3 mm-aperture) and EL-10-30 (10 mm-aperture) lenses, voice-coil actuation enables focal length tuning between −77 and 77 mm, and between 50 and 120 mm, respectively. Their maximum driving current is 120 and 400 mA, respectively, and their maximum power consumption is 100 and 2000 mW, respectively. Their rise times in response to a current step (time to reach 90% of the response) are ∼1 and 4 ms, respectively, with oscillations that settle within ∼4 and 15 ms, respectively (Optotune, 2021).

A less compact design, where the electro-magnetic chamber and the lens chamber were not concentric, was described by (Yu et al., 2011).

Electro-magnetic driving has also been implemented in a different way, where the fluid-filled reservoir is squeezed by an external servo motor; this solution was proposed by (Ren et al., 2006) and is currently commercialized by the company Holochip (Holochip, 2021).

In general, electromagnetic driving of fluid-based lenses advantageously offers large tuning ranges. However, it leads to devices that are relatively thick, heavy and energy inefficient. Moreover, as for other liquid-based lenses, they are also limited by sensitivity to thermal fluctuations, such that open-loop driving requires a temperature sensor to enable compensations (Optotune, 2021).

#### Dielectric Elastomer Actuation

Another technology used to pump the fluid with a compact architecture is represented by DE actuation. For instance, a structure similar to that described above for electromagnetic driving can be used, by creating a cylindrical actuation chamber closed by a fluid-pressurized annular DE actuator, as schematically presented in Figure 5C. Electrical charging of the electrodes causes their expansion and so moves the fluid out of the concentric lens chamber, thereby reducing the lens membrane’s curvature. Using that configuration, Wei et al. (2014) described a 5 mm-aperture lens (see Figure 5C’) capable of a focal length tuning from 25 to 105 mm at 1 kV and a response time of the order of 100 ms.

An alternative design, where the DE actuation chamber was placed aside (rather than concentrically with) the lens chamber, was described for an array of lenses by Niklaus et al. (2010) and for a single lens by Lau et al. (2014).

A more compact structure was described by Li et al. (2019b), still using concentric chambers, but removing the stiff separator shown in Figure 5C; however, performance was reduced (focal length variations up to 32% at 5 kV), due to both the lack of a constraint at the inner edge of the annular DE actuation membrane, and a reduction of the membrane size.

The concept in Figure 5C was also modified by Cheng et al. (2020), with an actuation chamber made of a thin toroidal DE actuator, consisting of an elastomeric encapsulation of the fluid, sandwiched between two annular compliant electrodes; this configuration however was limited by a larger separation between the DE electrodes, which required higher driving voltages (up to 12 kV for a focal length decrease of ∼500 mm with a 6 mm aperture).

In general, such lenses with variable fluid volumes displaced by DE actuation share most of the pros (large apertures, small thickness and low weight) and cons (high voltages) of DE actuation-driven encapsulated-fluid lenses, although here the response speed is typically lower, due to the time needed to displace the fluid.

#### Electrostrictive Polymer Actuation

Essentially the same configuration shown in Figure 5C has also been studied with electrostrictive polymer (EP) actuators. In particular, Choi et al. (2011) created, around a 2.4 mm-aperture lens chamber, four actuation chambers, having a PDMS membrane coupled to a multi-layered EP actuator. The latter consisted of a stack of 1.2 μm-thick poly(vinylidene fluoride-trifluoroethylene-chlorotrifluoroethylene) – P(VDF-TrFE-CTFE) copolymer films, coated with aluminum electrodes. Each actuator was able to push the fluid (inverse operation to that shown in Figure 5C), such that the focal length changed from ∼120 to 10 cm at 40 V, with a response time of ∼20 ms.

As compared to DE actuators (typically made of thermosetting polymers, such as PDMS elastomers), in general EP actuators can more easily be fabricated as one-order-of-magnitude thinner films (as they are made up of polymers that are thermoplastic and stiffer). As a result, EP actuators-based lenses can be operated at one-order-of-magnitude lower driving voltages. However, the electrically induced thickness strains of EPs are typically one-order-of-magnitude smaller than those of DEs (1% against at least 10%), thereby limiting the deformability of the actuation chamber. As a consequence, comparable focal length tuning ranges can only be achieved with smaller apertures.

#### Piezoelectric Actuation

Alternatively to the use of DE and EP actuators, a compact mechanism similar to that of Figure 5C can also be implemented with bending piezoelectric actuators. An example is schematically shown in Figure 5D, where such transducers compress the membrane of an actuation chamber, concentric with a lens chamber. This concept was described by Schneider et al. (2008), demonstrating a 5 mm-aperture lens (see Figure 5D’), with a focal length variation from 500 to 30 mm at 44 V.

In general, piezoelectric actuators advantageously require voltages lower than those necessary for DE actuators and comparable to those for EP actuation. Nevertheless, as their electrically induced strains are also lower (order of 0.1%), they need to be shaped as cantilever benders, in order to magnify the displacements. As a consequence of a limited deformability of the actuation chamber, piezoelectric-based lenses are usually limited to small apertures, in order to maintain adequate variations of the lens curvature.

#### Electrostatic Zipping Actuation

In addition to DE, EP and piezoelectric actuation, another electrostatic technology, called zipping actuation, has recently been described for hydraulic driving of fluid-based lenses. The zipping effect, which has been used for micro-electro-mechanical systems for at least the past 3 decades (Branebjerg and Gravesen, 1992), has also been implemented for macroscopic actuation of soft membranes (Maffli et al., 2013). Recently, its application to electrode-coated polymeric chambers filled in with dielectric liquids has led to new kinds of actuators, proposed for soft robotics (Kellaris et al., 2018) and tactile displays (Leroy et al., 2020). They have also been applied to tune the focal length of oil-filled lenses, according to the concept described in Figure 5E: electrical charging of metalized plastic membranes creates attraction between them, resulting in a progressive closure (zipping effect) of their initial angle and a concomitant displacement of the interposed insulating fluid; as a result, the curvature of a transparent elastomeric membrane in the lens chamber is increased (Hartmann et al., 2020). Figure 5E’ shows a prototype lens having a 6 mm aperture and a 16 mm total diameter (including the actuation part); its focal length was reported to vary between 550 and 22 mm at 500 V, with a response time of 260 ms (Hartmann et al., 2020).

The main limitations of this approach currently are the large size of the actuation area relative to the optical area, and the need for high voltages (as compared to electrostrictive or piezoelectric actuation-based lenses), although there are opportunities for a possible reduction by one order of magnitude (Hartmann et al., 2020).

#### Electro-Thermal Actuation

Hydraulically shaped lenses have also been proposed with electro-thermal driving. Figure 5F shows a possible strategy, based on the thermal expansion of a temperature-sensitive optical fluid, which increases the curvature a soft membrane that seals the fluid chamber. An example was described by Ashtiani and Jiang (2013), who developed a water-filled 2 mm-aperture lens (see Figure 5F’) capable of a focal length variation from 82 to 29 mm at 30°C, with reported rise time of 0.8 s and fall time of 1.4 s.

A modified version was proposed by Zhang et al. (2011), using a chamber similar to that in Figure 5F, where the thermally sensitive fluid was replaced with a passive one; the latter was displaced by a thermal expansion of a soft chamber, consisting of an air-filled heated cavity, closed by a PDMS membrane. The displacement of the fluid caused a decrease of the focal length of a 2 mm-aperture lens from 15 to 3 mm at 37°C, with a heating time of ∼50 s and a (passive) cooling time of ∼115 s.

A different strategy is presented in Figure 5G. Here, the thermal deformation of a temperature-sensitive polymer (such as a gel) is used to displace a fluid, which is interfaced to another immiscible fluid, via a meniscus that changes its curvature and so its focal length. This concept was implemented with an hydrogel by Dong et al. (2006), achieving for a 500 μm-aperture lens (see Figure 5G’) a focal length change from 11.7 to ∼50 mm at 33°C, with response times of ten to a few tens of seconds.

In general, whilst electro-thermally activated lenses can advantageously offer significant tuning ranges and low driving voltages, their main drawbacks are the size/weight and, especially, the power consumption of the heating system, as well as the long response times for heating and cooling. Moreover, the lens apertures appear to be limited both by the need for reducing the volume of the fluid (to reduce its thermal inertia) and/or by a limited thermal expansion of the fluid/polymer.

#### Other Types of Actuation

In addition to the main electro-(magneto/thermo)-mechanical pumping systems described above, also other kinds of energy transduction mechanisms have been proposed to drive hydraulically shaped lenses. Two examples are mentioned below.

The first one is a study by Xu et al. (2009), who used a bending actuator made of a photo-sensitive polymer, to be deformed by a control light at specific wavelengths (such as UV light); such electro-opto-mechanical strategies are however limited by a typically slow response speed of photo-deformable polymers (Xu et al., 2009).

The second example is a study by Qian et al. (2020), who achieved a displacement of a dielectric liquid (silicone) as a result of an electrostatic pressure generated via a corona charging of the liquid-air interface; whilst this approach allowed for a demonstration of a 3 mm-aperture lens with a focal length change from 10 to 30 mm at ∼6 kV, it was limited by the need for high driving voltages, which typically characterize any corona charging process.

Additional examples are not covered by this Review for the sake of brevity and many others are expected to come in the future, as basically any kind of actuation technology could be used to pressurize a fluid, in order to change the curvature of a deformable refractive surface. Indeed, this appears to be the easiest way to obtain a tunable lens.

## Fully Elastomeric Tunable Lenses

As described, fluid lenses are very popular, as in many cases it is rather easy to obtain broad tuning ranges. Nevertheless, a straightforward comparison between basic features of fluid lenses and, as a possible alternative, fully elastomeric (such as made of silicone) lenses, shows the following shortcomings of the former: 1) they are more sensitive to gravitational sagging and vibrations, especially as the aperture increases; 2) their surface shape is typically restricted to a (quasi-)spherical cap; 3) their fluid is sensitive to thermal fluctuations. Therefore, the possibility of making a fully elastomeric lens electrically tunable is attractive. Nevertheless, it is straightforward to recognize that the main advantages (higher mechanical and thermal stability, and customisable shape) are typically at the expense of having a lower tuning range, due to their higher stiffness.

The most relevant strategies to electrically tune the curvature of elastomeric lenses are reviewed below.

### Electrically Shaped Elastomeric Lenses

#### Electrically Sensitive Gel Actuation

**FIGURE 6:**
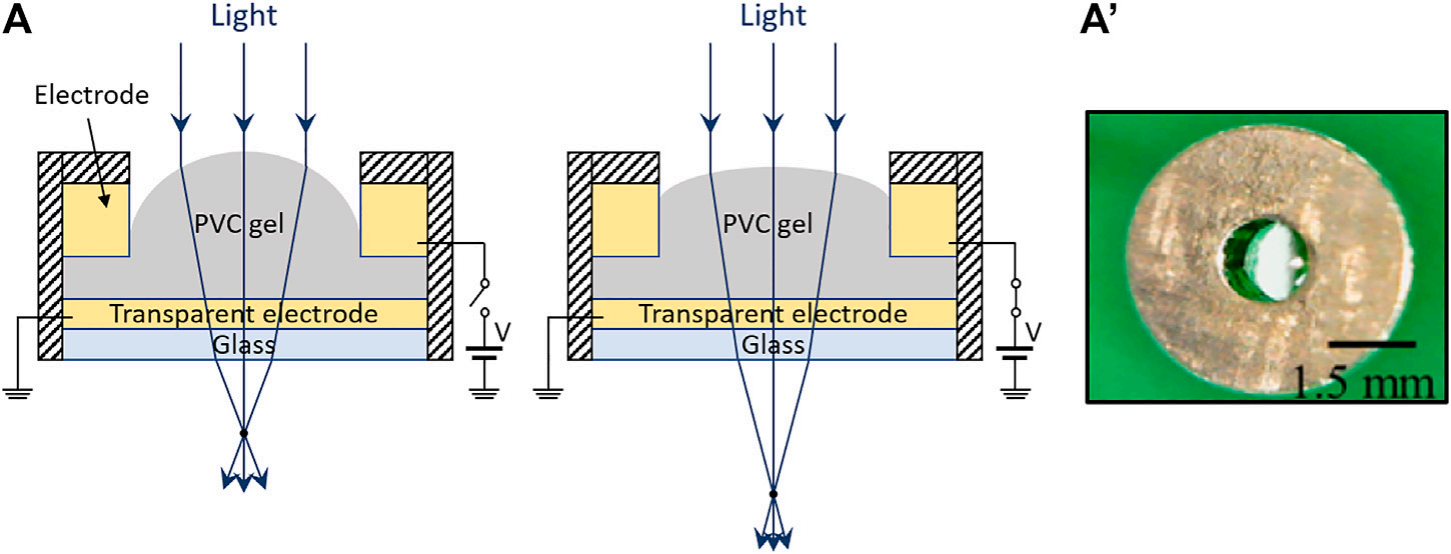
Electrically deformed elastomeric lens, consisting of an electrically sensitive PVC gel. **(A)** Schematic of a possible structure (example among various alternatives) and related principle of operation: an applied voltage creates a non-uniform electric field, which deforms the lens-shaped block of gel, changing its curvature. **(A′)** Prototype sample, reproduced with permission from (Bae et al., 2017).

A variety of electroactive polymers can show large electrically induced deformations (Carpi, 2016). Among them, Poly(vinyl chloride) - PVC gel combined with plasticizers has been studied over the past 2 decades (Uddin et al., 2001). By applying an electric field via two electrodes in contact with a plasticized PVC gel, the material deforms, as a result of an attraction toward the positively charged electrode; the effect is considered to originate from electrically induced orientations of polarized plasticizer molecules and dipole rotations of PVC chains (Ali et al., 2011). This phenomenon has been used to create tunable lenses. Moving from the first demonstration of the occurring optical effect (Hirai et al., 2009), tunable lenses have been configured as represented in Figure 6A. A plasticized PVC gel is confined between an annular electrode and a transparent flat electrode, so that an applied voltage can reduce an initial curvature of the gel surface, thereby increasing its focal length. A prototype sample is shown in Figure 6A’.

As an example, the focal length of 1.5 mm-aperture lenses was reported to vary either from less than 3.8–14.3 mm at 800 V (Kim et al., 2015), or from 5 to 15 mm at 500 V (Bae et al., 2015), or from 3 to 24.5 mm at 400 V (Choi et al., 2017). Microscopic lenses with an aperture of 300 µm were described by Lan et al. (2019), reporting a focal length variation from 2.75 to 3.15 mm at 250 V.

In order to achieve tunable biconvex lenses, the design shown in Figure 6A was modified, by sandwiching the plasticized PVC gel between two pairs of annular electrodes (Choi et al., 2017). These concepts have been implemented by plasticizing PVC with various molecules, such as acetyl tributyl citrate (Bae et al., 2015), dibutyl adipate (Choi et al., 2017) and dibutyl phthalate (Lan et al., 2019), and it is likely that new materials will be explored in the future to improve the electro-mechano-optical performance.

Due to the dielectric and mechanical losses of the constitutive material, the main drawback of this technology is its low response time, typically of the order of 1 s (Bae et al., 2015; Bae et al., 2017; Choi et al., 2017). However, microscopic versions of such lens can show higher speeds (Lan et al., 2019).

Another limitation lays in the fact that, with the design in Figure 6A, the deformation is sustained by a fringe electric field, at the edge of the lens, which therefore might limit the maximum possible aperture; nevertheless, this aspect should be confirmed by future investigations on lenses larger than 1.5 mm, which to date is the maximum diameter tested, to the best of our knowledge.

### Electro-Mechanically Shaped Elastomeric Lenses

#### External Motor Actuation

**FIGURE 7:**
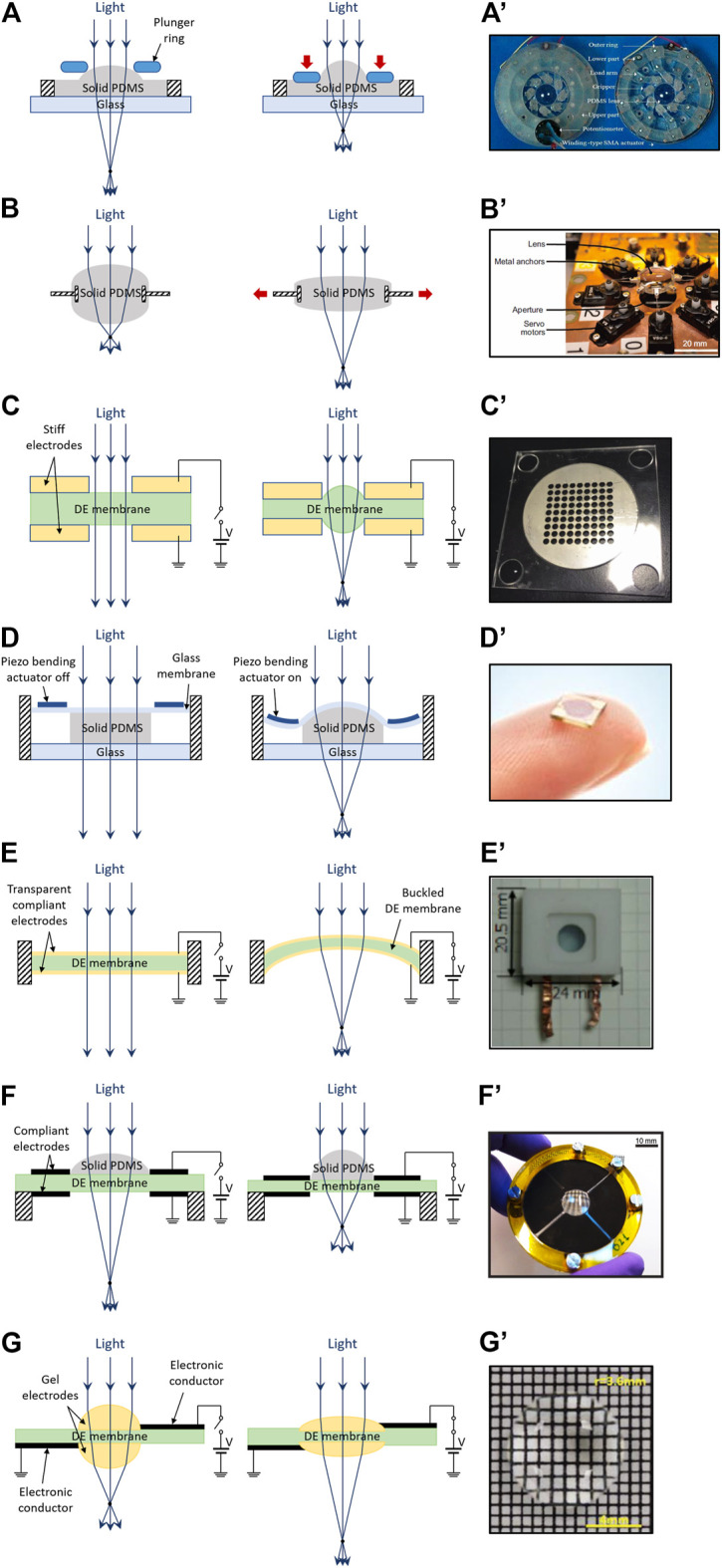
Electro-mechanically shaped elastomeric lenses. Structures and principles of operation **(left)** and photos of examples **(right)**, for different actuation strategies: **(A)** schematic of a lens squeezed by an external plunger ring; **(A′)** shape-memory-alloy-actuated prototype sample, reproduced with permission from (Choi et al., 2009); **(B)** schematic of a lens pulled by radial extenders; **(B′)** servo-motors-actuated prototype sample, reproduced with permission from (Liebetraut et al., 2013); **(C)** schematic of a lens operated by an electrostatic actuator, consisting of stiff annular electrodes that squeeze a soft membrane; **(C′)** array of prototype samples, reproduced with permission from (Wang et al., 2017); **(D)** schematic of a lens operated by piezoelectric bending actuators, which deform a thin glass membrane acting on the lens; **(D′)** commercial product by poLight, adapted from (Polight, 2021); **(E)** schematic of a lens operated by a transparent DE actuator, which forms the whole lens and increases its own curvature by buckling (note: unidirectional buckling should be facilitated by an initial asymmetry); **(E′)** prototype sample, reproduced with permission from (Son et al., 2012). **(F)** schematic of a lens radially compressed by an annular DE actuator; **(F′)** prototype sample, reproduced with permission from (Ghilardi et al., 2019); **(G)** schematic of a lens made of and radially stretched by transparent gel electrodes of a DE actuator; **(G′)** prototype sample, reproduced with permission from (Liu et al., 2020).

The most straightforward way to increase the curvature of an elastomeric lens is to squeeze it with an external motor. For instance, in Figure 7A a plunger ring is pushed onto a PDMS lens, causing a central bulging (Beadie et al., 2008).

Various types of actuators can be used to that purpose. An example is shown in Figure 7A’, where a biconvex PDMS lens was deformed by a mechanism driven by shape memory alloy actuators (Choi et al., 2009); an applied voltage of 3 V and a current of 2 A produced a focal length change from 16.8 to 18 mm, with a response time of the order of 1 s.

Another straightforward approach to change the curvature of a soft biconvex lens is to radially stretch it, as shown in Figure 7B. As for the previous case, a wide range of actuators can be used to achieve this. For instance, in Figure 7B’ a PDMS lens with embedded metallic anchors was deformed using an array of radially arranged servo motors, producing a focal length increase from ∼32 to ∼35 mm (Liebetraut et al., 2013). This strategy also allowed for a demonstration of a lens with mechanically tunable astigmatism along multiple directions and a response time of the order of 1 s (Liebetraut et al., 2013).

In general, such approaches are limited by the size, weight and power consumption of the motors. Moreover, depending on the design, motors can concentrate forces on small areas of a soft lens, generating non-uniform stresses and strains; this can lead to significant aberrations and/or limit the maximum deformability (and, so, the tuning range) in order to preserve the local integrity of the polymer.

#### Electrostatic Actuation

Compact actuation systems for elastomeric lenses can be obtained using the electrostatic effect shown in Figure 7C: a soft elastomeric membrane is coupled to a pair of annular stiff (metallic) electrodes, such that an electrically induced attraction between them causes a bulging of the elastomeric material in the central part, generating a bi-convex lens. By using this approach, Wang et al. (2017) demonstrated 1 mm-aperture lenses (Figure 7C’) capable of a focal length change from ∼1,000 cm (optical infinity) to 9.5 cm at 5 kV.

Whilst this solution attractively enables large tuning ranges with a compact and lightweight structure, it is challenged by the high voltages required by the electrostatic effect. Moreover, the aperture is limited by the elastomer’s actual deformation, which, for any given elastic modulus, is constrained by the need for minimizing the membrane’s thickness, to avoid excessive voltages.

#### Piezoelectric Actuation

As an additional type of electrostatic technology, piezoelectric driving is also used for elastomeric lenses, as sketched in Figure 7D. The lens consists of a deformable transparent polymeric body, sandwiched between a rigid glass substrate and a flexible (thin) glass membrane. The structure is deformed by a piezoelectric bending actuator ring. In particular, the bending motion causes the thin glass membrane to squeeze the polymer at the edge, so that the central part bulges upwards, forming a (plano-) convex lens.

Such lenses are commercially available under the name of TLens® (Figure 7D’) produced by the company poLight (Polight, 2021); they have apertures of ∼1 mm, are driven at ∼40 V and change their focal length from optical infinity to 10 cm, with a response time of ∼1 ms (Polight, 2021).

As already discussed for its use with fluid lenses, in general piezoelectric driving of elastomeric lenses offers fast responses, high control accuracies and low power consumption. As a drawback, the small electrically induced strains of piezoelectric materials, combined with the stiffness of the glass membrane-elastomeric body couple, limit the deformability of the latter; as a result, in order to ensure adequate variations of the lens curvature, this technology is limited to small apertures (smaller than those of piezoelectric-based liquid lenses).

#### Dielectric Elastomer Actuation

As a third example of electrostatic technology, DE actuation has also been applied to elastomeric lenses, according to the following three main strategies.

A first approach is represented in Figure 7E. A DE membrane coated with transparent compliant electrodes acts as a refractive structure when it buckles in response to an applied voltage; as the curvature is dependent on the voltage, a variable focal length is achieved. This concept was proposed by Son et al. (2012), who used a PDMS membrane with PEDOT transparent electrodes (see Figure 7E’).

A second strategy is shown in Figure 7F. It consists of a plano-convex PDMS lens arranged on the inner circular region of an annular DE actuator; upon electrical activation, the actuator radially squeezes the lens, which therefore bulges, reducing its focal length. This concept was first described by Pieroni et al. (2016), who created (by mold casting) a customizable PDMS lens directly on the DE membrane; a 12 mm-aperture lens showed a focal length change from 36.6 to 16.6 mm at 3.7 kV. This design was modified by Nam et al. (2018), to implement a stretching (instead of compression) of the lens, by arranging it on a parallel plane and connecting it to the actuation membrane via plastic couplers. The combination of the approach by Pieroni et al. (2016) with a segmentation of the electrodes was used by Ghilardi et al. (2019) to enable an electrical deformability of the lens along selectable directions (see Figure 7F’), so as to electrically control its astigmatism. The design by Pieroni et al. (2016) was also used by Park et al. (2020), who demonstrated a focal length tuning from ∼8.4 to ∼4.3 mm at 5 kV, with a response time of ∼7 ms.

A third strategy is shown in Figure 7G. It is based on a DE membrane coated on each side with a transparent, conductive and soft gel, shaped as a spherical cap, which is used both as a half-lens and as an actuation electrode; by electrically charging the two half-lenses/electrodes, the resulting surface expansion (of both the membrane and the electrodes) causes a reduction of the lens curvature. This approach was proposed by Liu et al. (2020), who demonstrated hydrogel-based lenses (Figure 7G’) having an aperture in the range 1–10 mm, capable of a focal length variation from 12.3 to 22.1 mm at 5.5 kV, with a response time of ∼153 ms.

In general, using DE actuation for elastomeric lenses shows most of the same pros (large apertures, small thickness and low weight) and cons (high voltages) of its use for fluid-based lenses. However, here additional advantages come from the solid state of the lens (as discussed in general above), although the downside typically is a lower tuning range, due to a higher stiffness.

### Electro-Thermally Shaped Elastomeric Lenses

#### Electro-Thermal Actuation

**FIGURE 8:**
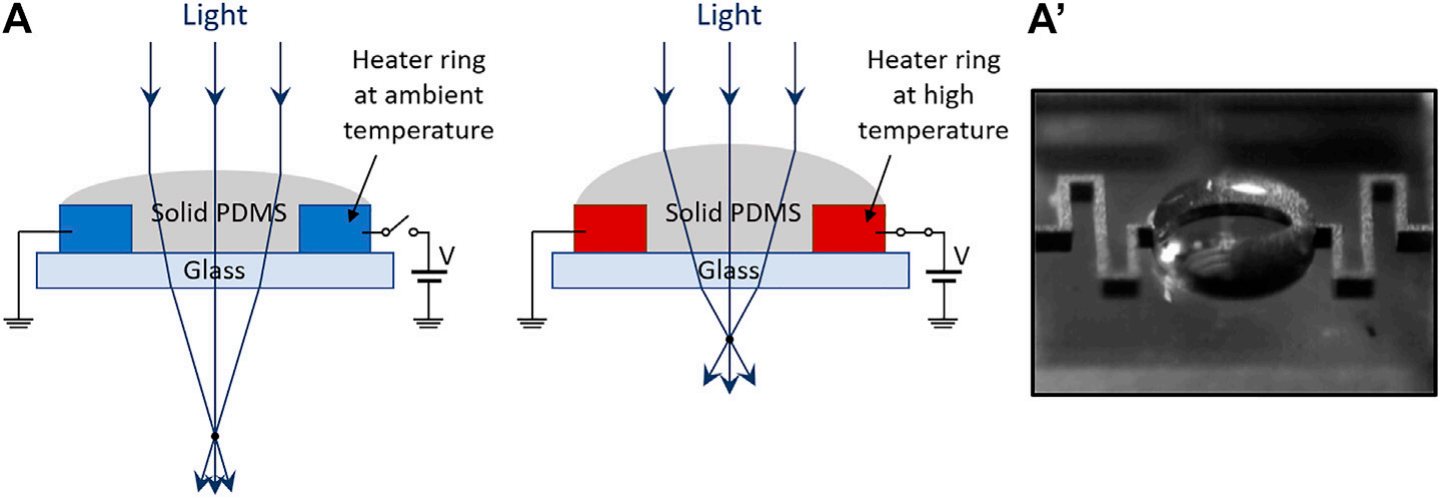
Electro-thermally shaped elastomeric lens: **(A)** Structure (example among various alternatives) and related principle of operation: an electro-thermal expansion of the polymeric lens increases its curvature; **(A′)** prototype sample, reproduced with permission from (Lee et al., 2007).

Elastomeric lenses can also be deformed with electro-thermal driving. Figure 8A shows a possible configuration, based on the thermal expansion of a polymer, pre-shaped as a lens-like spherical cap, arranged onto a circular heater. As an example, Lee et al. (2006) described a 200 μm-aperture PDMS lens showing a focal length decrease from 1852 to 1,018 μm, by driving the heater with a current of 70 mA, which led to a lens temperature of ∼325°C. That lens was modified by Lee et al. (2007) with an elliptical heater (see Figure 8A’), to demonstrate tunable astigmatism along one direction.

In general, electro-thermal actuation of elastomeric lenses shows, as for fluid-based lenses, the same pros (low driving voltages) and cons (size/weight and power consumption of the heater, long response times and small apertures). However, here the advantages deriving from the solid nature of the lens come at the expense of higher driving temperatures and lower tuning ranges.

## Comparisons Among Technologies

The different types of lenses for focal length tuning are compared in Table 1, summarizing the orders of magnitude of the most relevant indicators of performance, according to the data reported in the preceding sections.

**TABLE 1:** Comparison of the considered types of lenses. Each metric (except for the power consumption) is quantified in terms of orders of magnitude of typical values (abbreviations: +ve = positive; −ve = negative). For each lens, the most typical order of magnitude of the driving voltage is emphasized in bold. The power consumption is expressed qualitatively, as information published in different studies is often not comparable.

Tunable lens technology	Focal length variation		Voltage (V)	Resp. Time (ms)	Power cons	Aperture (mm)
*Liquid Crystal Lenses:*	From +∞	to +ve	**10**–100	10–100	Low	10
*Electrically Shaped Meniscus Lenses:*						
Electrowetting effect	From +ve	to +∞	**10**–100	10–100	Low	1–10
	From −∞	to −ve				
Dielectrophoretic effect	From +ve	to 10–100% lower	**10**–100	100–1,000	Low	1–10
*Electro-Mechanically Shaped Meniscus Lenses:*						
Dielectric elastomer actuation	From +ve	to 10–100% lower	100–**1,000**	—	Low	1–10
*Electro-Mechanically Shaped Encapsulated-Fluid Lenses:*						
Piezoelectric actuation	From +∞	to +ve	**100**	0.1–10	Low	1–10
Dielectric elastomer actuation	From +ve	to 10–100% lower	**1,000**	0.1–10	Low	10
	From +ve	to 10–100% higher				
*Hydraulically Shaped Lenses:*						
External pump actuation	From −ve	to −∞	1–**10**	1,000	High	10
	From +∞	to +ve				
Electromagnetic actuation	From −ve	to −∞	1–**10**–100	1–1,000	High	10
	From +∞	to +ve				
Dielectric elastomer actuation	From +ve	to 10–100% higher	100–**1,000**	100	Low	10
Electrostrictive polymer actuation	From +ve	to 100% lower	10–**100**	10	Low	1–10
Piezoelectric actuation	From +ve	to 100% lower	10–**100**	—	Low	1–10
Electrostatic zipping actuation	From +ve	to 100% lower	100–**1,000**	100	Low	10
Electro-thermal actuation	From +ve	to 10–100% lower	1–**10**	>1,000	High	1
	From +ve	to +∞				
	From −∞	to −ve				
*Electrically Shaped Elastomeric Lenses:*						
Electrically-sensitive gel actuation	From +ve	to 100–1,000% higher	**100**–1,000	1,000	Low	1
*Electro-Mechanically Shaped Elastomeric Lenses:*						
External motor actuation	From +ve	to 10% lower	1–**10**	1,000	High	10
	From +ve	to 10% higher				
Electrostatic actuation	From +∞	to +ve	**1,000**	—	Low	1
Piezoelectric actuation	From +∞	to +ve	10–**100**	1	Low	1
Dielectric elastomer actuation	From +∞	to +ve	**1,000**	100	Low	10
	From +ve	to 10–100% lower				
*Electro-Thermally Shaped Elastomeric Lenses:*						
Electro-thermal actuation	From +ve	to 10–100% lower	1–**10**	>1,000	High	0.1

It is worth noting that in Table 1, as well as across the whole text, the focusing performance is expressed in terms of range of focal length, rather than range of optical power. As the latter can easily be calculated (as the reciprocal) from the former, we deemed useful to provide explicit quantifications of the focal lengths achievable from state-of-the-art lenses, especially in view of practical needs for applications. Nevertheless, attention should be paid to the fact that the focal length is dependent on several other figures of merit, including aperture, volume/weight, total thickness and acceptable aberrations. As those other quantities are typically highly variable across different lenses reported in the literature, the focal length ranges are not fully comparable, in rigorous terms. The same consideration applies to the other metrics listed in Table 1.

Therefore, the table should only be used to compare common distinctive features of lenses currently available, either as research prototypes or commercial products. Conversely, it cannot be used to compare all the potentialities and limitations of the various technologies. These are discussed within the text, with an approach aimed at guiding non-expert readers, who then might refer to more specialized publications for more insights.

## Challenges for the Future

### Integration of Tunable Focusing With Tunable Zooming

Each strategy described above allows for varying the lens’ focal length; nevertheless, it cannot independently modulate also the lens’ magnification, as the two properties are coupled. The most straightforward way to make the focal length and magnification independently controllable is to use a combination of independently tunable lenses. This has been demonstrated, for instance, using electrowetting lenses (Li et al., 2017).

However, in order to reduce the size and weight of the system, a more attractive solution would come from the possibility of integrating tunable focusing and tunable zooming into a single lens. So far, very few attempts to combine those two degrees of freedom have been reported. As an example, this has been demonstrated in modified versions of electrowetting lenses, where a liquid column’s height was independently modulated for zooming, by displacing the fluid from a coaxial chamber, either via electromagnetic actuation (Park et al., 2018) or via electrowetting (Li et al., 2018).

Achieving such integration with simple and compact solutions, in order to exploit all the potential advantages of tunable lenses, is an important challenge for the future of this field.

### Independent Tunability of Multiple Aberrations

Another challenge, possibly even more important, is represented by an electrical controllability of different aberrations, in addition to focusing. As for the integration of focusing with zooming discussed above, the most straightforward approach is to use a combination of independently tunable lenses. This has been done, for instance, with hydraulic driving of two independently controllable lens chambers (Waibel et al., 2011).

However, the key challenge is to make multiple aberrations independently tunable (in the visible range) on a single lens. This aims not only to improve the quality of images, but also to simplify the implementation of optical functions that currently require complex systems (sets of multiple lenses). As an example, multi-directional control of astigmatism has been demonstrated with external motors (Liebetraut et al., 2013), piezoelectric actuation (Bonora et al., 2015), the electrowetting effect (Kopp and Zappe, 2016) and dielectric elastomer actuation (Ghilardi et al., 2019). Much remains to be done in order to complement focusing with independent control of various aberrations, which otherwise can only vary in a dependent way (Ghilardi et al., 2019; Peng et al., 2020; Bonora et al., 2015).

Developing such multi-functional lenses requires electrical deformability according to multiple degrees of freedom, thereby challenging the current state of the art of the enabling actuation technologies.

### Study of Case Scenarios to Design New Cameras for Robotic and Machine Vision

As recalled in the Introduction, varifocal lenses are expected to become increasingly relevant for future generations of cameras in various fields, such as consumer electronics, optical instrumentation, drones and wearable virtual/augmented-reality systems. Such applications can have very different requirements, in terms of several figures of merit of the lenses, including size, weight, focal range, aperture, speed, aberrations, driving voltage and power consumption. Therefore, the design of new cameras requires, firstly, systematic studies of key “case scenarios”, which should outline specifications and practical needs. These can be lacking to a large extent for those contexts of application that are still relatively new and, so, poorly explored, such as wearable virtual/augmented-reality systems. In such contexts, new uses are continuously emerging, setting new requirements. Depending on the application, in some cases current tunable lenses might already be practically usable, whilst, in other cases, greater potentialities offered by less mature technologies might encourage further developments.

## Concluding Remarks

A significant variety of electrically tunable lenses has been demonstrated so far, in order to simplify mechanisms for focusing and zooming in dynamic vision systems. This article has reviewed them, showing similarities and differences, and highlighting advantages and drawbacks of each approach.

Although some of them have become commercially available, no single strategy appears today as able to combine all the ideal requirements, namely compact size, low weight, mechanical and thermal stability, customisable shape, large tuning range, fast response, large aperture, low driving voltage and low power consumption.

We hope that this systematic Review might help to stimulate further research on tunable multi-functional lenses, capable to open up new opportunities for robotic and machine vision.

## Data Availability

The original contributions presented in the study are included in the article/Supplementary Material, further inquiries can be directed to the corresponding author.

## References

[B1] Agarwal M. Gunasekaran R. A. Coane P. Varahramyan K. (2004). Polymer-based Variable Focal Length Microlens System. J. Micromech. Microeng. 14, 1665–1673. 10.1088/0960-1317/14/12/010

[B2] Aieta F. Kats M. A. Genevet P. Capasso F. (2015). Multiwavelength Achromatic Metasurfaces by Dispersive Phase Compensation. Science 347, 1342–1345. 10.1126/science.aaa2494 25700175

[B3] Ali M. Ueki T. Tsurumi D. Hirai T. (2011). Influence of Plasticizer Content on the Transition of Electromechanical Behavior of PVC Gel Actuator. Langmuir 27, 7902–7908. 10.1021/la2009489 21604779

[B4] Allen M. (2014). Liquid crystal Lenses to Make Phones More Efficient. Horizon - the EU Research & Innovation magazine. online.

[B5] Almoallem Y. D. Jiang H. (2017). Double-sided Design of Electrodes Driving Tunable Dielectrophoretic Miniature Lens. J. Microelectromech. Syst. 26, 1122–1131. 10.1109/JMEMS.2017.2711966 29606846 PMC5875936

[B6] Ashtiani A. O. Jiang H. (2013). Thermally Actuated Tunable Liquid Microlens with Sub-second Response Time. Appl. Phys. Lett. 103, 111101. 10.1063/1.4820772

[B7] Bae J. W. Shin E.-J. Jeong J. Choi D.-S. Lee J. E. Nam B. U. (2017). High-performance Pvc Gel for Adaptive Micro-lenses with Variable Focal Length. Sci. Rep. 7, 2068. 10.1038/s41598-017-02324-9 28522844 PMC5437028

[B8] Bae J. W. Yeo M. Shin E.-J. Park W.-H. Lee J. E. Nam B.-U. (2015). Eco-friendly Plasticized Poly(vinyl Chloride)-Acetyl Tributyl Citrate Gels for Varifocal Lens. RSC Adv. 5, 94919–94925. 10.1039/c5ra15304b

[B9] Beadie G. Sandrock M. L. Wiggins M. J. Lepkowicz R. S. Shirk J. S. Ponting M. (2008). Tunable Polymer Lens. Opt. Express 16, 11847–11857. 10.1364/OE.16.011847 18679457

[B10] Beam Co (2021). Online: Availble at: www.beamco.com (Accessed May 26, 2021).

[B11] Berge B. Peseux J. (2000). Variable Focal Lens Controlled by an External Voltage: an Application of Electrowetting. Eur. Phys. J. E 3, 159–163. 10.1007/s101890070029

[B12] Blog O. (2019). Half Dome Updates: FRL Explores More Comfortable, Compact VR Prototypes for Work. Online: Available at: www.oculus.com/blog/half-dome-updates-frl-explores-more-comfortable-compact-vr-prototypes-for-work (Accessed September 25, 2019).

[B13] Bonora S. Jian Y. Zhang P. Zam A. Pugh E. N. Zawadzki R. J. (2015). Wavefront Correction and High-Resolution *In Vivo* OCT Imaging with an Objective Integrated Multi-Actuator Adaptive Lens. Opt. Express 23, 21931–21941. 10.1364/OE.23.021931 26368169 PMC4646522

[B14] Branebjerg J. Gravesen P. (1992). “A New Electrostatic Actuator Providing Improved Stroke Length and Force,” in Proc. IEEE Micro Electro Mech. Sys. Workshop Travemunde, Travemunde, Germany, February 4–7, 1992, 6–11.

[B15] Carpi F. Frediani G. Turco S. De Rossi D. (2011). Bioinspired Tunable Lens with Muscle-like Electroactive Elastomers. Adv. Funct. Mater. 21, 4152–4158. 10.1002/adfm.201101253

[B16] Chen B. Lu J. J. Yang C. H. Yang J. H. Zhou J. Chen Y. M. (2014). Highly Stretchable and Transparent Ionogels as Nonvolatile Conductors for Dielectric Elastomer Transducers. ACS Appl. Mater. Inter. 6, 7840–7845. 10.1021/am501130t 24758275

[B17] Chen H.-S. Wang Y.-J. Chang C.-M. Lin Y.-H. (2015). A Polarizer-free Liquid crystal Lens Exploiting an Embedded-Multilayered Structure. IEEE Photon. Technol. Lett. 27, 899–902. 10.1109/LPT.2015.2399932

[B18] Chen W. T. Zhu A. Y. Sanjeev V. Khorasaninejad M. Shi Z. Lee E. (2018). A Broadband Achromatic Metalens for Focusing and Imaging in the Visible. Nat. Nanotech 13, 220–226. 10.1038/s41565-017-0034-6 29292382

[B19] Cheng C.-C. Andrew Yeh J. (2007). Dielectrically Actuated Liquid Lens. Opt. Express 15, 7140–7145. 10.1364/OE.15.007140 19547032

[B20] Cheng X. Yu M. Ma J. Li B. Zhang Y. Wang P. (2020). An Entirely Soft Varifocal Lens Based on an Electro-Hydraulic Actuator. Smart Mater. Struct. 29, 045017. 10.1088/1361-665X/ab72e8

[B21] Chiu C.-P. Chiang T.-J. Chen J.-K. Chang F.-C. Ko F.-H. Chu C.-W. (2012). Liquid Lenses and Driving Mechanisms: a Review. J. Adhes. Sci. Tech. 26, 1773–1788. 10.1163/156856111X600514

[B22] Choi D.-S. Jeong J. Shin E.-J. Kim S.-Y. (2017). Focus-tunable Double Convex Lens Based on Non-ionic Electroactive Gel. Opt. Express 25, 20133–20141. 10.1364/OE.25.020133 29041697

[B23] Choi J.-M. Son H.-M. Lee Y.-J. (2009). Biomimetic Variable-Focus Lens System Controlled by Winding-type SMA Actuator. Opt. Express 17, 8152–8164. 10.1364/OE.17.008152 19434147

[B24] Choi S. T. Lee J. Y. Kwon J. O. Lee S. Kim W. (2011). Varifocal Liquid-Filled Microlens Operated by an Electroactive Polymer Actuator. Opt. Lett. 36, 1920–1922. 10.1364/OL.36.001920 21593935

[B25] Dong L. Agarwal A. K. Beebe D. J. Jiang H. (2006). Adaptive Liquid Microlenses Activated by Stimuli-Responsive Hydrogels. Nature 442, 551–554. 10.1038/nature05024 16885981

[B27] Dynamic Optics (2021). Online: Available at:. www.dynamic-optics.eu (Accessed May 26, 2021).

[B28] Carpi F. (2016). in Electromechanically Active Polymers: A Concise Reference (Zurich: Springer).

[B29] Gao K. Cheng H.-H. Bhowmik A. K. Bos P. J. (2015). Thin-film Pancharatnam Lens with Low F-Number and High Quality. Opt. Express 23, 26086–26094. 10.1364/03.23.02608610.1364/oe.23.026086 26480123

[B30] Ghilardi M. Boys H. Török P. Busfield J. J. C. Carpi F. (2019). Smart Lenses with Electrically Tuneable Astigmatism. Sci. Rep. 9, 16127. 10.1038/s41598-019-52168-8 31695061 PMC6834852

[B31] Hartmann F. Penkner L. Danninger D. Arnold N. Kaltenbrunner M. (2020). Soft Tunable Lenses Based on Zipping Electroactive Polymer Actuators. Adv. Sci. 8, 2003104. 10.1002/advs.202003104 PMC7856880 33552870

[B32] Hasan N. Banerjee A. Kim H. Mastrangelo C. H. (2017). Tunable-focus Lens for Adaptive Eyeglasses. Opt. Express 25, 1221–1233. 10.1364/OE.25.001221 28158006 PMC5772464

[B33] Hendriks B. H. W. Kuiper S. Van As M. A. J. Renders C. A. Tukker T. W. (2005). Electrowetting-based Variable-Focus Lens for Miniature Systems. Opt. Rev. 12, 255–259. 10.1007/s10043-005-0255-z

[B34] Himax (2021). Online: Available at: www.himax.com.tw/products/electrically-tunable-focusing-lenses/continuous-lens (Accessed May 26, 2021).

[B35] Hirai T. Ogiwara T. Fujii K. Ueki T. Kinoshita K. Takasaki M. (2009). Electrically Active Artificial Pupil Showing Amoeba-like Pseudopodial Deformation. Adv. Mater. 21, 2886–2888. 10.1002/adma.200802217

[B36] Holochip (2021). Online: Available at; www.holochip.com (Accessed May 26, 2021).

[B37] Huang H. Zhao Y. (2019). Optofluidic Lenses for 2D and 3D Imaging. J. Micromech. Microeng. 29, 073001. 10.1088/1361-6439/ab1999

[B38] Jablonowski M. (2020). Beyond Drone Vision: the Embodied Telepresence of First-Person-View Drone Flight. Senses Soc. 15, 344–358. 10.1080/17458927.2020.1814571

[B39] Jamali A. Bryant D. Bhowmick A. K. Bos P. J. (2020). Large Area Liquid crystal Lenses for Correction of Presbyopia. Opt. Express 28, 33982–33993. 10.1364/OE.408770 33182876

[B40] Jin B. Ren H. Choi W.-K. (2017). Dielectric Liquid Lens with Chevron-Patterned Electrode. Opt. Express 25, 32411–32419. 10.1364/OE.25.032411

[B41] Kellaris N. Gopaluni Venkata V. Smith G. M. Mitchell S. K. Keplinger C. (2018). Peano-HASEL Actuators: Muscle-Mimetic, Electrohydraulic Transducers that Linearly Contract on Activation. Sci. Robot. 3, eaar3276. 10.1126/scirobotics.aar3276 33141696

[B42] Keplinger C. Sun J.-Y. Foo C. C. Rothemund P. Whitesides G. M. Suo Z. (2013). Stretchable, Transparent, Ionic Conductors. Science 341, 984–987. 10.1126/science.1240228 23990555

[B43] Kim S.-Y. Yeo M. Shin E.-J. Park W.-H. Jang J.-S. Nam B.-U. (2015). Fabrication and Evaluation of Variable Focus and Large Deformation plano-convex Microlens Based on Non-ionic Poly(vinyl Chloride)/dibutyl Adipate Gels. Smart Mater. Struct. 24, 115006. 10.1088/0964-1726/24/11/115006

[B44] Kopp D. Zappe H. (2016). Tubular Astigmatism-Tunable Fluidic Lens. Opt. Lett. 41, 2735–2738. 10.1364/OL.41.002735 27304276

[B45] Krogmann F. Mönch W. Zappe H. (2006). A MEMS-Based Variable Micro-lens System. J. Opt. A: Pure Appl. Opt. 8, S330–S336. 10.1088/1464-4258/8/7/S06

[B46] Kuiper S. Hendriks B. H. W. (2004). Variable-focus Liquid Lens for Miniature Cameras. Appl. Phys. Lett. 85, 1128–1130. 10.1063/1.1779954

[B47] Kumar M. B. Kang D. Jung J. Park H. Hahn J. Choi M. (2020). Compact Vari-Focal Augmented Reality Display Based on Ultrathin, Polarization-Insensitive, and Adaptive Liquid crystal Lens. Opt. Lasers Eng. 128, 106006. 10.1016/j.optlaseng.2020.106006

[B48] Lan C. Zhou Z. Ren H. Park S. Lee S. H. (2019). Fast-response Microlens Array Fabricated Using Polyvinyl Chloride Gel. J. Mol. Liquids 283, 155–159. 10.1016/j.molliq.2019.03.050

[B49] Lau G.-K. La T.-G. Shiau L.-L. Tan A. W. Y. (2014). Challenges of Using Dielectric Elastomer Actuators to Tune Liquid Lens. Proc. SPIE 9056, 90561J. 10.1117/12.2046384

[B50] Lee S.-Y. Chen W.-C. Tung H.-W. Fang W. (2007). Microlens with Tunable Astigmatism. IEEE Photon. Technol. Lett. 19, 1383–1385. 10.1109/LPT.2007.903010

[B51] Lee S.-y. Tung H.-w. Chen W.-c. Fang W. (2006). Thermal Actuated Solid Tunable Lens. IEEE Photon. Technol. Lett. 18, 2191–2193. 10.1109/LPT.2006.883891

[B52] LensVector (2021). Online: Available at: www.lensvector.com (Accessed May 26, 2021).

[B53] Leroy E. Hinchet R. Shea H. (2020). Multimode Hydraulically Amplified Electrostatic Actuators for Wearable Haptics. Adv. Mater. 32, 2002564. 10.1002/adma.202002564 32700326

[B54] Levy U. Shamai R. (2008). Tunable Optofluidic Devices. Microfluid Nanofluid 4, 97–105. 10.1007/s10404-007-0216-x

[B55] Li C. Jiang H. (2012). Electrowetting-driven Variable-Focus Microlens on Flexible Surfaces. Appl. Phys. Lett. 100, 231105. 10.1063/1.4726038 22904571 PMC3382254

[B56] Li J. Wang Y. Liu L. Xu S. Liu Y. Leng J. (2019b). A Biomimetic Soft Lens Controlled by Electrooculographic Signal. Adv. Funct. Mater. 29, 1903762. 10.1002/adfm.201903762

[B57] Li L.-Y. Yuan R.-Y. Wang J.-H. Li L. Wang Q.-H. (2019a). Optofluidic Lens Based on Electrowetting Liquid Piston. Sci. Rep. 9, 13062. 10.1038/s41598-019-49560-9 31506551 PMC6736858

[B58] Li L. Wang J.-H. Wang Q.-H. Wu S.-T. (2018). Displaceable and Focus-Tunable Electrowetting Optofluidic Lens. Opt. Express 26, 25839–25848. 10.1364/OE.26.025839 30469679

[B59] Li L. Yuan R.-Y. Wang J.-H. Wang Q.-H. (2017). Electrically Optofluidic Zoom System with a Large Zoom Range and High-Resolution Image. Opt. Express 25, 22280–22291. 10.1364/OE.25.022280 29041541

[B60] Liang D. Lin Z. F. Huang C. C. Shih W. P. (2014). Tunable Lens Driven by Dielectric Elastomer Actuator with Ionic Electrodes. Micro Nano Lett. 9, 869–873. 10.1049/mnl.2014.0401

[B61] Liebetraut P. Petsch S. Liebeskind J. Zappe H. (2013). Elastomeric Lenses with Tunable Astigmatism. Light Sci. Appl. 2, e98. 10.1038/lsa.2013.54

[B62] Lin H.-C. Lin Y.-H. (2010). A Fast Response and Large Electrically Tunable-Focusing Imaging System Based on Switching of Two Modes of a Liquid crystal Lens. Appl. Phys. Lett. 97, 063505. 10.1063/1.3479051

[B63] Lin Y.-H. Wang Y.-J. Reshetnyak V. (2017). Liquid crystal Lenses with Tunable Focal Length. Liquid Crystals Rev. 5, 111–143. 10.1080/21680396.2018.1440256

[B64] Liu S. Cheng D. Hua H. (2008). “An Optical See-Through Head Mounted Display with Addressable Focal Planes,” in Proc. IEEE Int. Symp. Mixed & Augm. Reality 2008, Cambridge, United Kingdom, September 15–18, 2008 (New York, NY: IEEE), 33–42.

[B65] Liu S. Qiu Y. Yu W. (2020). Self‐Contained Focus‐Tunable Lenses Based on Transparent and Conductive Gels. Macromol. Mater. Eng. 305, 2000393. 10.1002/mame.202000393

[B66] Maffli L. Rosset S. Ghilardi M. Carpi F. Shea H. (2015). Ultrafast All-Polymer Electrically Tunable Silicone Lenses. Adv. Funct. Mater. 25, 1656–1665. 10.1002/adfm.201403942

[B67] Maffli L. Rosset S. Shea H. R. (2013). Zipping Dielectric Elastomer Actuators: Characterization, Design and Modeling. Smart Mater. Struct. 22, 104013. 10.1088/0964-1726/22/10/104013

[B68] Mishra K. van den Ende D. Mugele F. (2016). Recent Developments in Optofluidic Lens Technology. Micromachines 7, 102. 10.3390/mi7060102 PMC6190348 30404276

[B69] Mugele F. Baret J.-C. (2005). Electrowetting: from Basics to Applications. J. Phys. Condens. Matter 17, R705–R774. 10.1088/0953-8984/17/28/R01

[B70] Nam S. Yun S. Yoon J. W. Park S. Park S. K. Mun S. (2018). A Robust Soft Lens for Tunable Camera Application Using Dielectric Elastomer Actuators. Soft Robotics 5, 777–782. 10.1089/soro.2017.0146 30156468

[B71] Naumov A. F. Loktev M. Y. Guralnik I. R. Vdovin G. (1998). Liquid-crystal Adaptive Lenses with Modal Control. Opt. Lett. 23, 992–994. 10.1364/OL.23.000992 18087406

[B72] Nguyen N.-T. (2010). Micro-optofluidic Lenses: a Review. Biomicrofluidics 4, 031501. 10.1063/1.3460392 20714369 PMC2921414

[B73] Niklaus M. Rosset S. Shea H. (2010). Array of Lenses with Individually Tunable Focal-Length Based on Transparent Ion-Implanted EAPs. Proc. SPIE 7642, 76422K. 10.1117/12.848445

[B74] Oh S. H. Rhee K. Chung S. K. (2016). Electromagnetically Driven Liquid Lens. Sensors Actuators A: Phys. 240, 153–159. 10.1016/j.sna.2016.01.048

[B75] Optotune (2021). Online: Available: www.optotune.com (Accessed May 26, 2021).

[B76] Park B. J. Park S. Choi M. Park S. K. Yun S. Shin E. (2020). Monolithic Focus-Tunable Lens Technology Enabled by Disk-type Dielectric-Elastomer Actuators. Sci. Rep. 10, 1–6. 10.1038/s41598-020-73666-0 33037237 PMC7547700

[B77] Park I. S. Park Y. Oh S. H. Yang J. W. Chung S. K. (2018). Multifunctional Liquid Lens for Variable Focus and Zoom. Sensors Actuators A: Phys. 273, 317–323. 10.1016/j.sna.2018.02.017

[B78] Pelrine R. Kornbluh R. Pei Q. Joseph J. (2000). High-speed Electrically Actuated Elastomers with Strain Greater Than 100%. Science 287, 836–839. 10.1126/science.287.5454.836 10657293

[B79] Pelrine R. E. Kornbluh R. D. Joseph J. P. (1998). Electrostriction of Polymer Dielectrics with Compliant Electrodes as a Means of Actuation. Sensors Actuators A: Phys. 64, 77–85. 10.1016/S0924-4247(97)01657-9

[B80] Peng T. Dai C. Lou J. Cui Y. Tao B. Ma J. (2020). A Low-Cost Deformable Lens for Correction of Low-Order Aberrations. Opt. Commun. 460, 125209. 10.1016/j.optcom.2019.125209

[B81] Pieroni M. Lagomarsini C. De Rossi D. Carpi F. (2016). Electrically Tunable Soft Solid Lens Inspired by Reptile and Bird Accommodation. Bioinspir. Biomim. 11, 065003. 10.1088/1748-3190/11/6/065003 27783568

[B82] Pohl H. A. (1978). Dielectrophoresis. Cambridge: Cambridge University Press.

[B83] Polight (2021). Online: Available at: www.polight.com (Accessed May 26, 2021).

[B84] Qian S. Shi W. Zheng H. Liu Z. (2020). Tunable-focus Liquid Lens through Charge Injection. Micromachines 11, 109. 10.3390/mi11010109 PMC7019498 31968568

[B85] Rasti P. Hous H. Schlaak H. F. Kiefer R. Anbarjafari G. (2015a). Dielectric Elastomer Stack Actuator-Based Autofocus Fluid Lens. Appl. Opt. 54, 9976–9980. 10.1364/AO.54.009976 26836566

[B86] Rasti P. Kesküla A. Haus H. Schlaak H. F. Anbarjafari G. Aabloo A. (2015b). A Passive Autofocus System by Using Standard Deviation of the Image on a Liquid Lens. Proc. SPIE 9430, 94301Q. 10.1117/12.2084198

[B87] Ren H. Fox D. Anderson P. A. Wu B. Wu S.-T. (2006). Tunable-focus Liquid Lens Controlled Using a Servo Motor. Opt. Express 14, 8031–8036. 10.1364/OE.14.008031 19529173

[B88] Ren H. Wu S.-T. (2012). Introduction to Adaptive Lenses. Hoboken, NJ: Wiley. 10.1002/9781118270080

[B89] Ren H. Xianyu H. Xu S. Wu S.-T. (2008). Adaptive Dielectric Liquid Lens. Opt. Express 16, 14954–14960. 10.1364/OE.16.014954 18795032

[B90] Sato S. (1979). Liquid-crystal Lens-Cells with Variable Focal Length. Jpn. J. Appl. Phys. 18, 1679–1684. 10.1143/jjap.18.1679

[B91] Schneider F. Müller C. Wallrabe U. (2008). A Low Cost Adaptive Silicone Membrane Lens. J. Opt. A: Pure Appl. Opt. 10, 044002. 10.1088/1464-4258/10/4/044002

[B92] She A. Zhang S. Shian S. Clarke D. R. Capasso F. (2018). Adaptive Metalenses with Simultaneous Electrical Control of Focal Length, Astigmatism, and Shift. Sci. Adv. 4, eaap9957. 10.1126/sciadv.aap9957 29507880 PMC5834009

[B93] Shian S. Clarke D. R. (2016). Electrically Tunable Window Device. Opt. Lett. 41, 1289–1292. 10.1364/OL.41.001289 26977691

[B94] Shian S. Diebold R. M. Clarke D. R. (2013). Tunable Lenses Using Transparent Dielectric Elastomer Actuators. Opt. Express 21, 8669–8676. 10.1364/OE.21.008669 23571956

[B95] Son S.-i. Pugal D. Hwang T. Choi H. R. Koo J. C. Lee Y. (2012). Electromechanically Driven Variable-Focus Lens Based on Transparent Dielectric Elastomer. Appl. Opt. 51, 2987–2996. 10.1364/AO.51.002987 22614602

[B96] Stevens R. Jacoby T. N. Aricescu I. S. Rhodes D. P. (2017). A Review of Adjustable Lenses for Head Mounted Displays. Proc. SPIE 10335, 103350Q. 10.1117/12.2276677

[B97] Supekar O. D. Zohrabi M. Gopinath J. T. Bright V. M. (2017). Enhanced Response Time of Electrowetting Lenses with Shaped Input Voltage Functions. Langmuir 33, 4863–4869. 10.1021/acs.langmuir.7b00631 28431469

[B98] Tabiryan N. V. Serak S. V. Roberts D. E. Steeves D. M. Kimball B. R. (2015). Thin Waveplate Lenses of Switchable Focal Length - New Generation in Optics. Opt. Express 23, 25783–25794. 10.1364/OE.23.025783 26480092

[B99] Uddin M. Z. Yamaguchi M. Watanabe M. Shirai H. Hirai T. (2001). Electrically Induced Creeping and Bending Deformation of Plasticized Poly(vinyl Chloride). Chem. Lett. 30, 360–361. 10.1246/cl.2001.360

[B100] Varioptic C. (2021). Online: Available at: www.varioptic.com (Accessed May 26, 2021).

[B101] Waibel P. Mader D. Liebetraut P. Zappe H. Seifert A. (2011). Chromatic Aberration Control for Tunable All-Silicone Membrane Microlenses. Opt. Express 19, 18584–18592. 10.1364/OE.19.018584 21935227

[B102] Wang L. Hayakawa T. Ishikawa M. (2017). Dielectric-elastomer-based Fabrication Method for Varifocal Microlens Array. Opt. Express 25, 31708–31717. 10.1364/OE.25.031708 29245842

[B103] Wang Y.-J. Lin Y.-H. (2019). An Optical System for Augmented Reality with Electrically Tunable Optical Zoom Function and Image Registration Exploiting Liquid crystal Lenses. Opt. Express 27, 21163–21172. 10.1364/OE.27.021163 31510198

[B104] Wapler M. C. (2020). Ultra-fast, High-Quality and Highly Compact Varifocal Lens with Spherical Aberration Correction and Low Power Consumption. Opt. Express 28, 4973–4987. 10.1364/OE.382472 32121727

[B105] Watson A. M. Dease K. Terrab S. Roath C. Gopinath J. T. Bright V. M. (2015). Focus-tunable Low-Power Electrowetting Lenses with Thin Parylene Films. Appl. Opt. 54, 6224–6229. 10.1364/AO.54.006224 26193397

[B106] Wei K. Domicone N. W. Zhao Y. (2014). Electroactive Liquid Lens Driven by an Annular Membrane. Opt. Lett. 39, 1318–1321. 10.1364/ol.39.001318 24690736

[B107] Werber A. Zappe H. (2005). Tunable Microfluidic Microlenses. Appl. Opt. 44, 3238–3245. 10.1364/AO.44.003238 15943257

[B108] Xu S. Ren H. Lin Y.-J. Moharam M. G. J. Wu S.-T. Tabiryan N. (2009). Adaptive Liquid Lens Actuated by Photo-Polymer. Opt. Express 17, 17590–17595. 10.1364/OE.17.017590 19907543

[B109] Xu S. Ren H. Wu S.-T. (2013). Dielectrophoretically Tunable Optofluidic Devices. J. Phys. D: Appl. Phys. 46, 483001. 10.1088/0022-3727/46/48/483001

[B110] Yang D.-K. Wu S.-T. (2014). Fundamentals of Liquid Crystal Devices. 2nd edition. Hoboken, NJ: Wiley. 10.1002/9781118751992

[B111] Yu H. Guo B. Tsu-Hui A. L. Lin J. T. M. Yee T. B. (2012). Characterization of the Dynamic Mechanical Stability of Liquid-Filled Lenses. Opt. Express 20, 23720–23727. 10.1364/OE.20.023720 23188337

[B112] Yu H. Zhou G. Chau F. S. Sinha S. K. (2011). Tunable Electromagnetically Actuated Liquid-Filled Lens. Sensors Actuators A: Phys. 167, 602–607. 10.1016/j.sna.2011.03.005

[B113] Zang J. Ryu S. Pugno N. Wang Q. Tu Q. Buehler M. J. (2013). Multifunctionality and Control of the Crumpling and Unfolding of Large-Area Graphene. Nat. Mater 12, 321–325. 10.1038/nmat3542 23334002 PMC3605241

[B114] Zappe H. Duppé C. (2016). Tunable Micro-optics. Cambridge: Cambridge University Press.

[B115] Zhang D.-Y. Lien V. Berdichevsky Y. Choi J. Lo Y.-H. (2003). Fluidic Adaptive Lens with High Focal Length Tunability. Appl. Phys. Lett. 82, 3171–3172. 10.1063/1.1573337

[B116] Zhang H. Ren H. Xu S. Wu S.-T. (2014). Temperature Effects on Dielectric Liquid Lenses. Opt. Express 22, 1930–1939. 10.1364/OE.22.001930 24515202

[B117] Zhang W. Aljasem K. Zappe H. Seifert A. (2011). Completely Integrated, Thermo-Pneumatically Tunable Microlens. Opt. Express 19, 2347–2362. 10.1364/OE.19.002347 21369053

[B118] Zou X. Zheng G. Yuan Q. Zang W. Chen R. Li T. (2020). Imaging Based on Metalenses. Photoni. X 1. 10.1186/s43074-020-00007-9

